# Supplementary material for: Expressed Protein Selenoester Ligation
Source: Angew Chem Int Ed Engl. 2022 Mar 9;61(20):e202200163. doi: 10.1002/anie.202200163 (PMC9314092; doi:10.1002/anie.202200163)
Supplement: Supplementary file 1 — Supporting Information [file ANIE-61-0-s001.pdf]

Angewandte Chemie
Eine Zeitschrift der Gesellschaft Deutscher Chemiker

Supporting Information

Expressed Protein Selenoester Ligation

S. S. Kulkarni, E. E. Watson, J. W. C. Maxwell, G. Niederacher, J. Johansen-Leete, S. Huhmann, S. Mukherjee, A. R. Norman, J. Kriegesmann, C. F. W. Becker, R. J. Payne**

Table of Contents

Materials	3
General peptide synthesis procedures	4
General procedure for acyl hydrazide to selenoester conversion	6
Ubiquitin acyl hydrazide to selenoester conversion	6
General procedure for one-pot selenoesterification, ligation and deselenization (EPSL)	11
Synthesis of Ubiquitin-TMEM43 conjugate	11
Synthesis of Ubiquitin-ZAP70 conjugate	14
Semi-synthesis of lipidated analogues of YPT6	18
Folding of semi-synthetic lipidated analogues of YPT6	28
Semi-synthesis of Hsp27 phosphoforms	30
Folding and CD spectra of Hsp27 phosphoforms	42
Chaperone assay for Hsp27 phosphoforms	43
^{1}H and ^{13}C NMR spectra (building blocks **S1** and **S2**)	45
References	49

Materials

Peptide grade dimethylformamide (DMF) was obtained from Labscan. Amino acids, coupling reagents and resins for Fmoc-solid-phase peptide synthesis (SPPS) were obtained from either Novabiochem, GL Biochem or Sigma Aldrich. Manual SPPS was performed in polypropylene syringes equipped with Teflon filters, purchased from Torviq. Analytical reversed-phase ultra-performance liquid chromatography (RP-UPLC) was performed on either a Waters Acquity UPLC system equipped with PDA eλ detector (λ = 210 – 400 nm), Sample Manager FAN and Quaternary Solvent Manager (H-class) modules or a Waters System 2695 separations module with a 2996 photodiode array detector. Peptides were analyzed using an XBridge BEH 5 µm, 2.1 x 150 mm wide-pore column (C18) at a flow rate of 0.7 mL min^{-1} or 1 mL min^{-1} on the HPLC system or Waters Acquity UPLC BEH 1.7 µm 2.1 x 50 mm column (C18) at a flow rate of 0.6 mL min^{-1} on the UPLC system. Gradients for the UPLC system were run over five minutes with an initial 1 min equilibration step (i.e. gradient from 1-6 min) while the gradients for the HPLC system were run for 30 min with an initial 5 min equilibration step (i.e. gradient from 5-35 min). Both instruments were run using a mobile phase composed of 0.1% trifluoroacetic acid (TFA) in H_2O (Solvent A) and 0.1% trifluoroacetic acid in acetonitrile (Solvent B) in a linear gradient as indicated. Analysis of the chromatograms was conducted using Empower 3 Pro software (2010) and retention times (R_t min) of pure peptides and proteins are reported with the gradients specified.

Preparative and semi-preparative reversed-phase HPLC was performed using a Waters 600E Multisolvent Delivery System with a Rheodyne 7725i injection valve (5 mL loading loop) and Waters 500 pump with a Waters 490E programmable wavelength detector operating at 214, 230, 254 or 280 nm. Preparative reversed-phase HPLC was performed using a Waters Sunfire C18 column (5 µm, 19 × 250 mm) at a flow rate of 14 mL min^{-1}. Semi-preparative reversed-phase HPLC was performed using either a Waters XBridge-BEH300 wide-pore C18 column (5 µm, 10 × 250 mm) or Symmetry C4 column (300 Å, 5 µm, 10 mm × 250 mm) at a flow rate of 4 mL min^{-1} or (300 Å, 5 µm, 2.1 mm × 150 mm) at a flow rate of 0.7 mL min^{-1}. Ubiquitin conjugate **5** was purified on a Phenomenex Luna C18 column (100 Å, 5 µm, 10 × 250 mm) heated to 50 °C using a Waters column heater module at a flow rate of 4 mL min^{-1}, with the gradients as described. A mobile phase of 0.1% trifluoroacetic acid in water (Solvent A) and 0.1% trifluoroacetic acid in acetonitrile (Solvent B) was used in all other cases, using the linear gradients specified. After lyophilization, peptides were isolated as trifluoroacetate salts.

LC-MS was performed either on a Shimadzu LC-MS 2020 instrument consisting of a LC-M20A pump and a SPD-20A UV/Vis detector coupled to a Shimadzu 2020 mass spectrometer (ESI) operating in positive mode, or a Shimadzu UPLC-MS equipped with the same modules as the LC-MS system except for a SPD-M30A diode array detector. Separations were performed on the LC-MS system either on a Waters Sunfire 5 µm, 2.1 x 150 mm column (C18), or wide-pore equivalent operating at a flow rate of 0.7 mL min^{-1} or 1 mL min^{-1}. Separations on the UPLC-MS system were performed using a Waters Acquity UPLC BEH 300Å 1.7 µm 2.1 x 50 mm column (C18) at a flow rate of 0.6 mL min^{-1}. Separations were performed using a mobile phase of 0.1% formic acid in water (Solvent A) and 0.1% formic acid in acetonitrile (Solvent B) and a linear gradient of 0-50% B over 30 min on the LC-MS System and 0-60% B over 8 min on the UPLC-MS system.

Low-resolution mass spectra were recorded on a Shimadzu 2020 mass spectrometer (ESI) operating in positive or negative mode as indicated. The deconvoluted mass spectra were generated using MagTran software.[1] Low Resolution MALDI-TOF mass spectra were recorded on a Bruker Autoflex™ Speed MALDI-TOF instrument operating in linear mode using a sinapinic acid matrix (10 mg/mL in water/acetonitrile (7:3 v/v) containing 0.1 vol. % TFA).

General peptide synthesis procedures

Fmoc-Strategy SPPS General Procedures (100-200 µmol scale)

Loading of Hydrazide-Functionalized Resin

2-Chlorotrityl chloride resin (0.5 g, 1.22 mmol/g loading) was swollen in dry CH_2Cl_2 for 30 min, followed by washing with CH_2Cl_2 (10 x 3 mL). A solution of 5 vol. % $N_2H_4{\cdot}H_2O$ in DMF (5 mL) was added to the resin and allowed to shake for 30 min at room temperature. The resin was then washed with DMF (5 x 3 mL), CH_2Cl_2 (5 x 3 mL) and DMF (5 x 3 mL), followed by a repeat treatment with 5 vol. % $N_2H_4{\cdot}H_2O$ in DMF (5 mL) and washing. A capping solution of CH_2Cl_2:MeOH:DIPEA (17:2:1, v/v/v, 5 mL) was then added to the resin and allowed to shake at room temperature for 40 min. The resin was then washed with CH_2Cl_2 (5 x 3 mL) and DMF (5 x 3 mL) to afford a light yellow-green resin. Fmoc-Xaa-OH (4 eq. relative to resin loading), PyBOP (4 eq. relative to resin loading) and N-methylmorpholine (8 eq. relative to resin functionalization) in DMF (final concentration 0.125 M of amino acid) was added to the resin, which was shaken at room temperature for 1 h. The resin was then washed with DMF (5 x 3 mL), CH_2Cl_2 (5 x 3 mL) and DMF (5 x 3 mL). The resin was then capped *via* treatment with 10 vol.% acetic anhydride in pyridine (5 mL) for 5 mins at room temperature. The resin was then washed again with DMF (5 x 3 mL), CH_2Cl_2 (5 x 3 mL) and DMF (5 x 3 mL) prior to determination of the estimated loading of the first amino acid.

Loading to Rink Amide Resin

Rink amide resin (0.6 mmol/g loading) was swollen in dry CH_2Cl_2 for 30 min, followed by washing with CH_2Cl_2 (5 x 3 mL) and DMF (5 x 3 mL). Fmoc-Xaa-OH, DIC and Oxyma (4.0:4.0:4.4 eq. relative to resin loading, respectively) in DMF (final concentration 0.125 M of amino acid) were added to the resin, which was shaken at room temperature for 1 h. The resin was then washed with DMF (5x 3 mL), CH_2Cl_2 (5x 3 mL) and DMF (5 x 3 mL). The resin was then capped *via* treatment with 10 vol.% acetic anhydride in pyridine (5 mL) for 5 mins at room temperature. The resin was then washed again with DMF (5x 3 mL), CH_2Cl_2 (5 x 3 mL) and DMF (5 x 3 mL) prior to determination of the estimated loading of the first amino acid.

Loading Estimation of the First Amino Acid

The resin was treated with piperidine/DMF (1:4 v/v, 3 mL, 2x 5 min) and then washed with DMF (5x 3 mL), CH_2Cl_2 (5x 3 mL) and DMF (5x 3 mL). The combined deprotection solutions were then made up to 10 mL with fresh piperidine/DMF (1:4 v/v). The solution was diluted 50-100 fold with fresh piperidine/DMF (1:4 v/v) and the UV absorbance of the piperidine-fulvene adduct

measured (λ = 301 nm, ε = 7800 $M^{-1}cm^{-1}$) to estimate the amount of amino acid loaded onto the resin.

Automated Synthesis of Model Peptides

Peptides were synthesized on a Biotage SYRO I automated peptide synthesizer according to the general setup below. Coupling solutions – Fmoc-protected amino acids (0.5 M in DMF), Oxyma (0.55 M in DMF) and DIC (0.5 M in DMF); Capping solution – 10 vol. % DIPEA and 5 vol. % acetic anhydride in DMF; Deprotection solution – 20 or 40 vol. % piperidine in DMF. A standard coupling synthesis program is as follows: 1 x 3 mL 40 vol. % piperidine in DMF, 5 min; drain; 1 x 3 mL 20 vol. % piperidine in DMF, DMF wash (4 x 5 mL); 5 mL 1.1:1:1 molar ratio Oxyma:DIC:AA in 4 equivalent excess over the resin, 50 °C, 30 min; 4 x 5 mL DMF wash; 1 x 3 mL acetic anhydride (5 vol. %) and DIPEA (10 vol. %) in DMF, 6 min; 4 x 5 mL DMF wash.

Coupling of Boc-Sec(PMB)-OH or $(Boc\text{-}Sec\text{-}OH)_2$

Boc-Sec(PMB)-OH or $(Boc\text{-}Sec\text{-}OH)_2$ amino acids[2] (1.2 eq.) were coupled using Oxyma (1.2 eq.) and DIC (1.2 eq.) in DMF (0.1 M) for 16 h at room temperature with Fmoc deprotection and capping conducted as described above for standard SPPS. In the case of peptides involving phosphorylated amino acids, HATU coupling conditions [Boc-Sec(PMB)-OH (2.5 eq.) or $(Boc\text{-}Sec\text{-}OH)_2$ (1.25 eq.), HATU (2.5 eq.), Oxyma (2.5 eq.) and DIPEA (7.5 eq.) in DMF (0.1 M) for 16 h at room temperature] were employed instead of DIC/Oxyma.

Coupling of phosphorylated Amino Acids [Fmoc-Thr(PO(OBzl)OH)-OH and Fmoc-Ser(PO(OBzl)OH)-OH]

Phosphorylated amino acids Fmoc-Thr(PO(OBzl)OH)-OH and Fmoc-Ser(PO(OBzl)OH)-OH (2.5 eq.) were coupled using HATU (2.5 eq.), Oxyma (2.5 eq.) and DIPEA (7.5 eq.) in DMF (0.1 M) for 16 h at room temperature with Fmoc deprotection and capping conducted as described above for standard SPPS.

Cleavage from the Resin

Resin-bound peptides were treated with a TFA:$i Pr_3SiH$:water mixture (90:5:5 v/v/v, 5 mL) and allowed to shake at room temperature for 2 h. For YPT6 peptides, ammonium iodide (20 eq.) was also added to the cleavage cocktail. At this point, the resin was filtered and washed with fresh cleavage cocktail. The combined cleavage solutions were worked-up as described below.

Work-up and Purification

Ice-cold Et_2O (40 mL) was added dropwise to the concentrated cleavage solution to precipitate the crude peptide. The precipitate was then collected *via* centrifugation and washed further with Et_2O to remove any remaining scavengers. Residual Et_2O was removed under gentle nitrogen flow and the pellet dissolved in 0.1% v/v TFA aqueous buffer (with minimal MeCN to aid dissolution, if necessary). The crude peptide was analysed by LC-MS (ESI) and purified by RP-HPLC.

General Procedure for PMB Deprotection

The PMB-protected selenopeptide was dissolved in a cocktail of TFA:DMSO:6 M Gn.HCl, 0.1 M HEPES buffer (3:1:1 v/v/v, 7 mg/mL peptide concentration) and the resulting solution was gently

agitated at room temperature. Upon complete PMB deprotection (as judged by UPLC-MS), the reaction mixture was diluted with water and purified by RP-HPLC to afford the corresponding peptide diselenide dimer.

General procedure for acyl hydrazide to selenoester conversion:

The following describes the general optimized procedure for selenoester formation under which all conversions were conducted unless otherwise stated.

A buffer solution containing 200 mM TCEP, 200 mM HEPES, 50 mM DPDS and 6 M Gnd.HCl was freshly prepared in MilliQ water and the pH was adjusted to 1.5-2.0 using 5 M aqueous HCl, followed by sparging of the solution with argon for 15-20 min. The peptide or protein acyl hydrazide substrate was then dissolved in the buffer (to final concentrations of 250 µM), followed by the addition of 5 eq. of acetylacetone (acac) from a 150 mM aqueous stock. The solution was then allowed to stir at room temperature under an atmosphere of argon for 2-3 h. Completion of the selenoesterification reaction was monitored by UPLC-MS followed by Et_2O extraction of residual DPDS. The crude reaction mixture was either subjected directly to a DSL reaction (in the case of the EPSL methodology) or subjected to HPLC purification and lyophilization to afford the purified peptide selenoester as a white fluffy solid.

Ubiquitin acyl hydrazide to selenoester conversion:

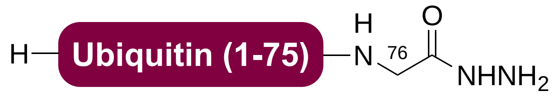

MQIFVKTLTGKTITLEVEPSDTIENVKAKIQDKEGIPPD
QQRLIFAGKQLEDGRTLSDYNIQKESTLHLVLRLRGG

Recombinant expression and purification of Ubiquitin-hydrazide:

The DNA sequence of human ubiquitin (Ub) was amplified by PCR (primer sequences attached) and subsequently cloned upstream of the *Mycobacterium xenopi* DNA Gyrase A (*Mxe* GyrA) intein, a His_7 tag and a chitin-binding domain (CBD) into a pTXB1 (New England Biolabs) plasmid via *Nde*I and *Spe*I restriction sites. Protein expression was performed using the *E. coli* BL21(DE3) Gold (Agilent) strain and 2YT medium (16 g/L Trypton, 10 g/L Yeast extract, 5 g/L NaCl) containing 100 µg/mL ampicillin at 37 °C.
Overnight cultures were diluted to OD_{600} 0.2, grown until OD_{600} 0.7 and protein overexpression was induced with 1 mM IPTG. After 2 h, the culture was centrifuged for 20 min at 10,000 g, the cell pellets were resuspended in TBS buffer (50 mM Tris, 150 mM NaCl, pH 8) and lysed twice

in a high-pressure cell disrupter (Constant Systems). The lysate was centrifuged for 30 min at 50,000 g. Each 45 mL supernatant was incubated with 5 mL TBS-equilibrated chitin resin (NEB) for 2 h. The resin was washed twice with 45 mL TBS and subsequently incubated with 30 mL hydrazine solution (5% hydrazine in TBS, 100 mM DTT) for 48 h to produce the Ub-hydrazide. After cleavage, the supernatant was separated from the chitin resin by centrifugation, filtered and purified by RP-HPLC on a C4 column (Kromasil C4, 300 Å, 10 µm, 21.2 mm × 250 mm; 5 to 30% B over 5 min then 30 to 70% B over 40 min, 0.1% v/v TFA, flow rate: 10 mL/min).

Ub-forward primer:
ATACATATGCAGATCTTCGTG

Ub-MxeSpe-reverse primer:
AATACTAGTGCATCTCCCGTGATGCAACCACCTCTGAGACGGAGTA

Ub sequence without intein:
MQIFVKTLTGKTITLEVEPSDTIENVKAKIQDKEGIPPDQQRLIFAGKQLEDGRTLSDYNI
QKESTLHLVLRLRGG

Mxe GyrA intein:

CITGDALVALPEGESVRIADIVPGARPNSDNAIDLKVLDRHGNPVLADRLFHSGEHPVYT
VRTVEGLRVTGTANHPLLCLVDVAGVPTLLWKLIDEIKPGDYAVIQRSAFSVDCAGFAR
GKPEFAPTTYTVGVPGLVRFLEAHHRDPDAQAIADELTDGRFYYAKVASVTDAGVQPV
YSLRVDTADHAFITNGFVSHATGLTGIHHHHHHSGLNSGLTTNPGVSAWQVNTAYTA
GQLVTYNGKTYKCLQPHTSLAGWEPSNVPALWQLQ

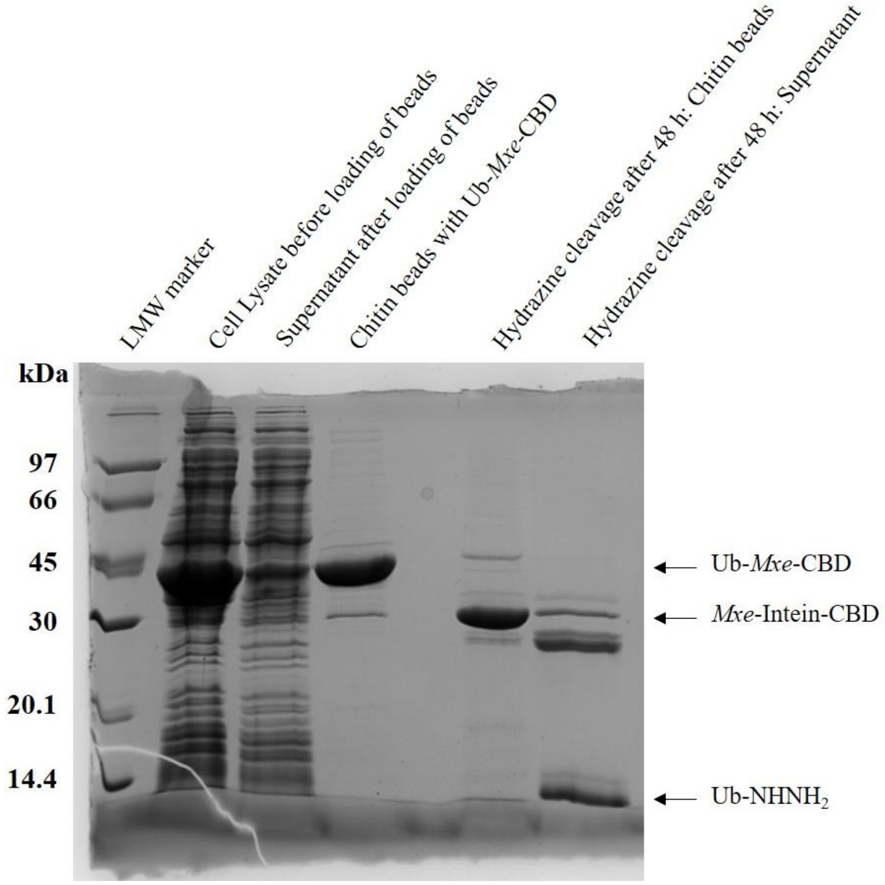

Figure S1: SDS-PAGE of Ub(1-76) cleavage from intein fusion construct with 5% hydrazine in the presence of 100 mM DTT. Intein-His_7-CBD remains bound to chitin beads and Ub(1-76)-$NHNH_2$ (MW ~8 kDa) is found in the supernatant.

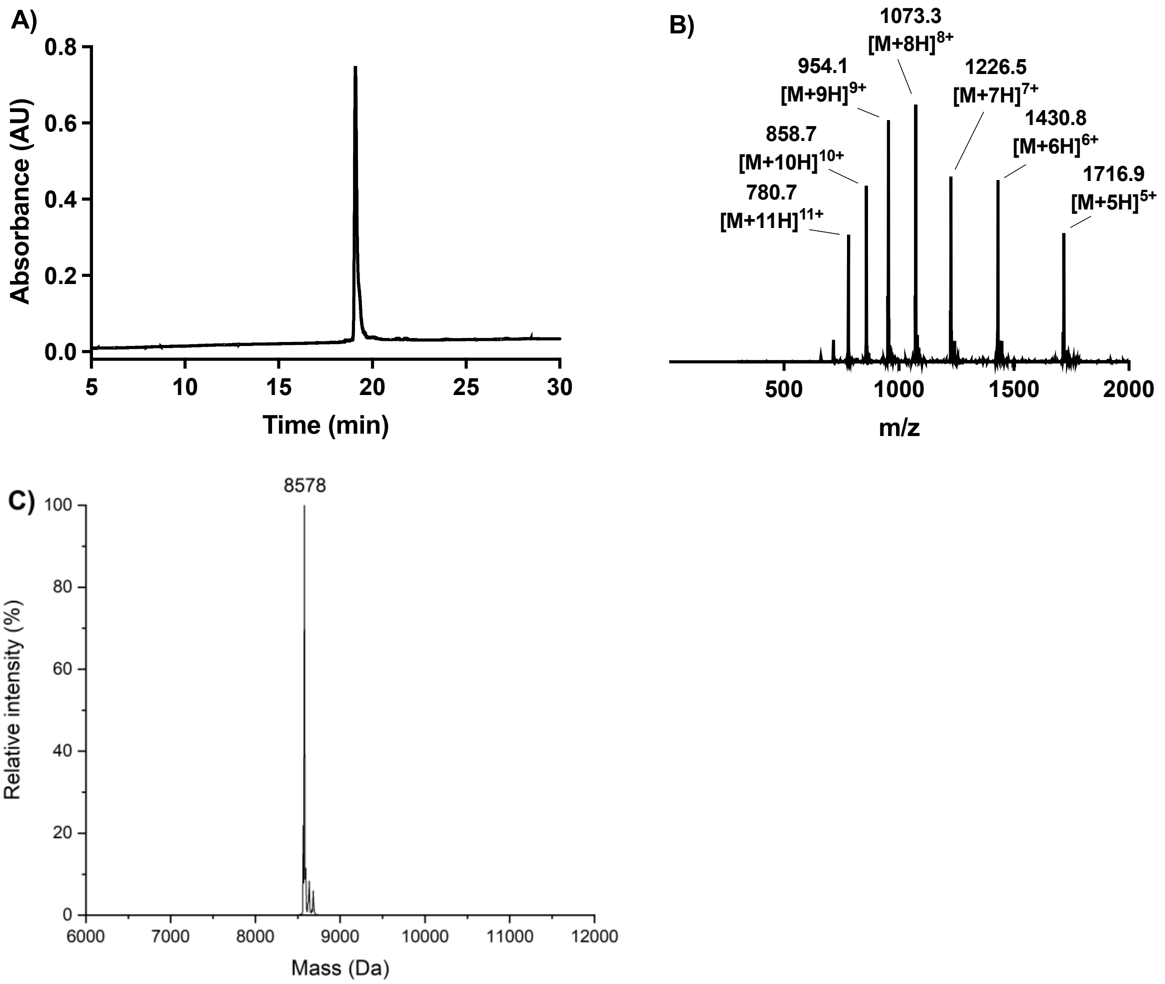

Figure S2: (A) Analytical HPLC trace of expressed Ubiquitin-$NHNH_2$ (**2**), R_t 19.1 min (1 to 50% B over 30 min, 0.1% v/v TFA, λ = 214 nm); **(B)** ESI-MS of pure **2**; Calculated Mass: $[M+5H]^{5+}$: 1716.8; $[M+6H]^{6+}$: 1430.8; $[M+7H]^{7+}$: 1226.6; $[M+8H]^{8+}$: 1073.4; $[M+9H]^{9+}$: 954.2; $[M+10H]^{10+}$: 858.9; $[M+11H]^{11+}$: 780.9. Mass Found (ESI+) 1716.9 $[M+5H]^{5+}$; 1430.8 $[M+6H]^{6+}$; 1226.5 $[M+7H]^{7+}$; 1073.3 $[M+8H]^{8+}$; 954.1 $[M+9H]^{9+}$; 858.7 $[M+10H]^{10+}$; 780.7 $[M+11H]^{11+}$. ESI-MS data was collected over the entire gradient and wash cycle of the UPLC-MS; **(C)** Deconvoluted mass spectrum of **2**.

Ubiquitin (1-76)-SePh

Recombinantly expressed **Ubiquitin (1-76)-$NHNH_2$** (2.0 mg, 0.21 μmol) was subjected to the selenoesterification conditions described in the general procedure (pH 2.0, 2.5 h, room temperature). Purification of the crude selenoester by preparative RP-HPLC (0 to 40% B over 40 min, 0.1% v/v TFA) afforded **Ubiquitin (1-76)-SePh** (**3**) as a fluffy white solid after lyophilization (1.2 mg, 57% yield).

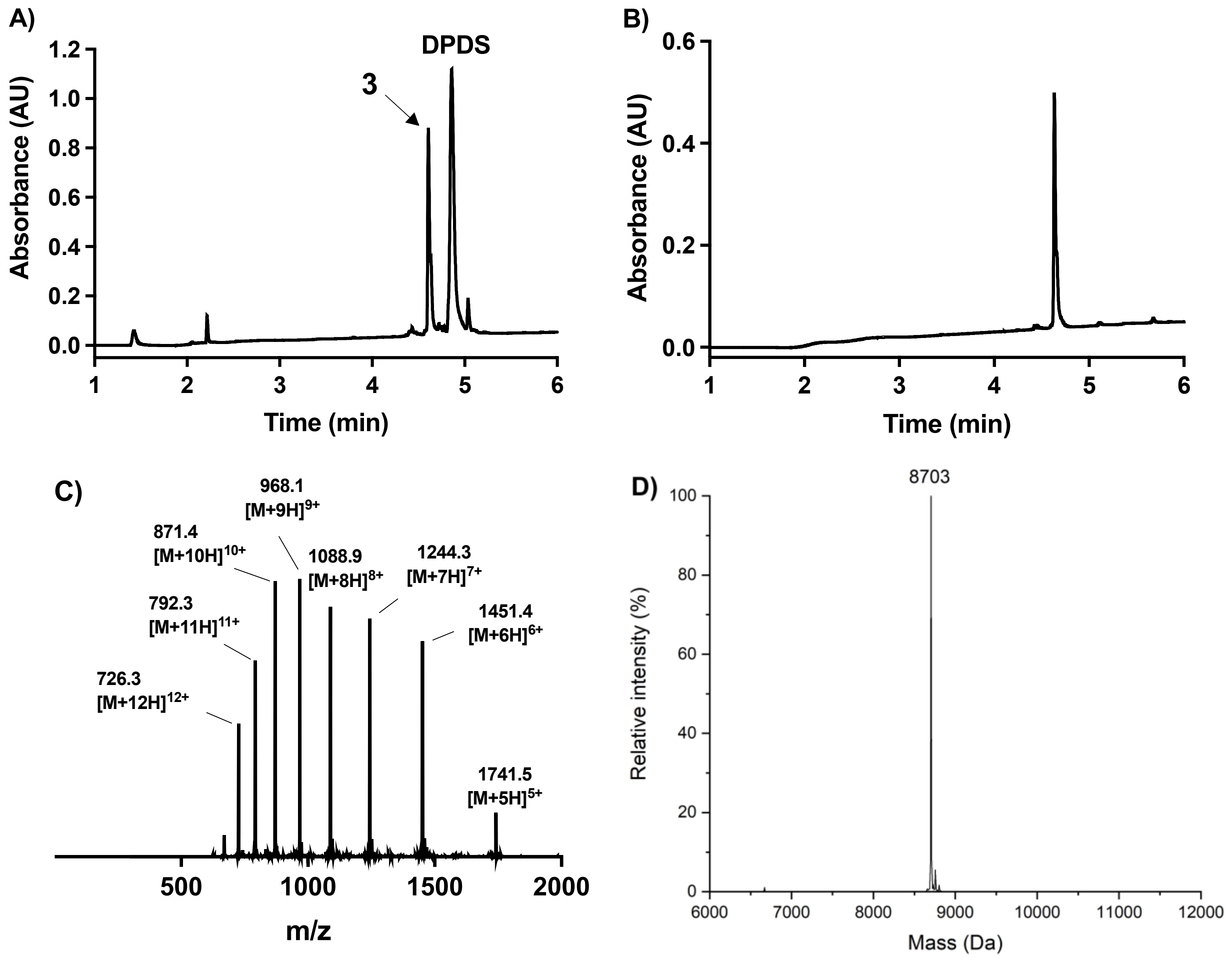

Figure S3: (A) Crude analytical HPLC trace of Ubiquitin (1-76)-SePh **(3)**; **(B)** Analytical HPLC trace of purified **3**, R_t 4.63 min (0 to 60% B over 5 min, 0.1% v/v TFA, λ = 214 nm) **(C)** ESI-MS of pure **3**; Calculated Mass: $[M+5H]^{5+}$: 1742.0; $[M+6H]^{6+}$: 1451.8; $[M+7H]^{7+}$: 1244.5; $[M+8H]^{8+}$: 1089.1; $[M+9H]^{9+}$: 968.2; $[M+10H]^{10+}$: 871.5; $[M+11H]^{11+}$: 792.3; $[M+12H]^{12+}$: 726.4. Mass Found (ESI+) 1741.5 $[M+5H]^{5+}$; 1451.4 $[M+6H]^{6+}$; 1244.3 $[M+7H]^{7+}$; 1088.9 $[M+8H]^{8+}$; 968.1 $[M+9H]^{9+}$; 871.4 $[M+10H]^{10+}$; 792.3 $[M+11H]^{11+}$; 726.3 $[M+12H]^{12+}$. ESI-MS data was collected over the entire gradient and wash cycle of the UPLC-MS; **(D)** Deconvoluted mass spectrum of **3**.

General procedure for one-pot selenoesterification, ligation and deselenization (EPSL)

The protein acyl hydrazide substrate was converted to the corresponding selenoester according to the general procedure above. The peptide diselenide dimer fragment was then added to the crude reaction mixture and the pH carefully raised to pH 5 using 5 M $NaOH_{(aq)}$ and the reaction was allowed to proceed at room temperature, with completion of the reaction assessed by UPLC-MS. Following completion of the ligation (typically in 40-50 min), Et_2O extraction (x10) was performed to remove residual DPDS. Solid TCEP (40 mM) and reduced glutathione (40 mM) were then added and the pH of resulting solution was adjusted to 7.0 followed by thorough sparging with argon. The reaction mixture was incubated at room temperature or 37 ^{o}C for 16 h as specified in the experimental procedure.

Synthesis of Ubiquitin-TMEM43 conjugate:

Synthesis of TMEM43(2-31) diselenide (4):

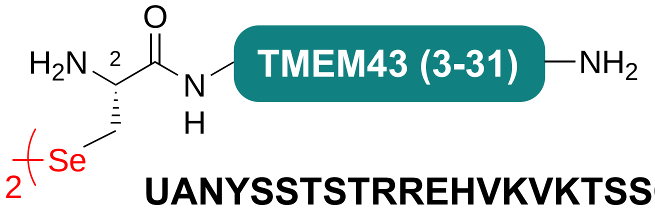

UANYSSTSTRREHVKVKTSSQPGFLERLSE

TMEM43 (2-31) was prepared according to Fmoc-strategy SPPS on Rink amide resin (50 µmol) as outlined in the general procedures, followed by coupling $(Boc\text{-}Sec\text{-}OH)_2$ at the N-terminus. Removal of the acid labile protecting groups, with concomitant cleavage from resin, was achieved *via* treatment with a solution of TFA/$i$$Pr_3SiH/H_2O$ (90:5:5 v/v/v, 10 mL) for 2 h at room temperature. After filtering off the resin, the deprotection solution was concentrated under nitrogen flow and the crude peptide was precipitated from ice-cold Et_2O. Purification of the crude peptide (25 µmol) by preparative RP-HPLC (0 to 50% B over 45 min, 0.1% v/v TFA) afforded diselenide **TMEM43 (2-31)** as a fluffy white solid after lyophilization (14.3 mg, 17% yield).

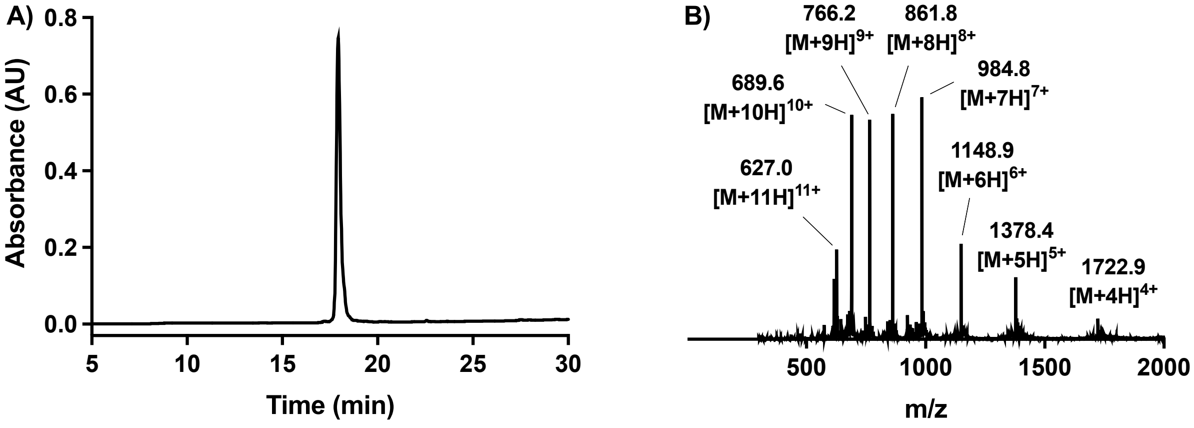

Figure S4: (A) Analytical HPLC trace of purified **TMEM43 (2-31) diselenide dimer** (**4**), R_t 17.9 min (0 to 70% B over 30 min, 0.1% v/v TFA, λ = 214 nm); **(B)** ESI-MS of pure **4**; Calculated Mass: $[M+4H]^{4+}$: 1722.9; $[M+5H]^{5+}$: 1378.5; $[M+6H]^{6+}$: 1148.9; $[M+7H]^{7+}$: 984.9; $[M+8H]^{8+}$: 861.9; $[M+9H]^{9+}$: 766.3; $[M+10H]^{10+}$: 689.7; $[M+11H]^{11+}$: 627.1. Mass Found (ESI+) 1722.9 $[M+4H]^{4+}$; 1378.4 $[M+5H]^{5+}$; 1148.9 $[M+6H]^{6+}$; 984.8 $[M+7H]^{7+}$; 861.8 $[M+8H]^{8+}$; 766.2 $[M+9H]^{9+}$; 689.6 $[M+10H]^{10+}$; 627.0 $[M+11H]^{11+}$. ESI-MS data was collected over the entire gradient and wash cycle of the UPLC-MS.

Ubiquitin-TMEM43(2-31) conjugate (5):

Ubiquitin (1-76)-NHNH₂ (2.3 mg, 0.23 µmol) was subjected to the selenoesterification conditions described in the general procedure, followed by the addition of **TMEM43(2-31)** (0.6 mg, 0.15 µmol) in accordance with the general procedure for ligation-deselenization described above. Due to the pH dependent solubility of TMEM43(2-31), both the ligation and deselenization steps were conducted at pH 3.4, with VA-044 (5 eq.) added to the deselenization to allow reaction completion within 16 h. Purification of the crude peptide by preparative RP-HPLC (0 to 20% B over 5 min then 20 to 50% B over 40 min at 50 °C, 0.1% v/v TFA) afforded the **Ubiquitin(1-76)-TMEM43(2-31)** conjugate as a fluffy white solid after lyophilization (1.3 mg, 62% yield).

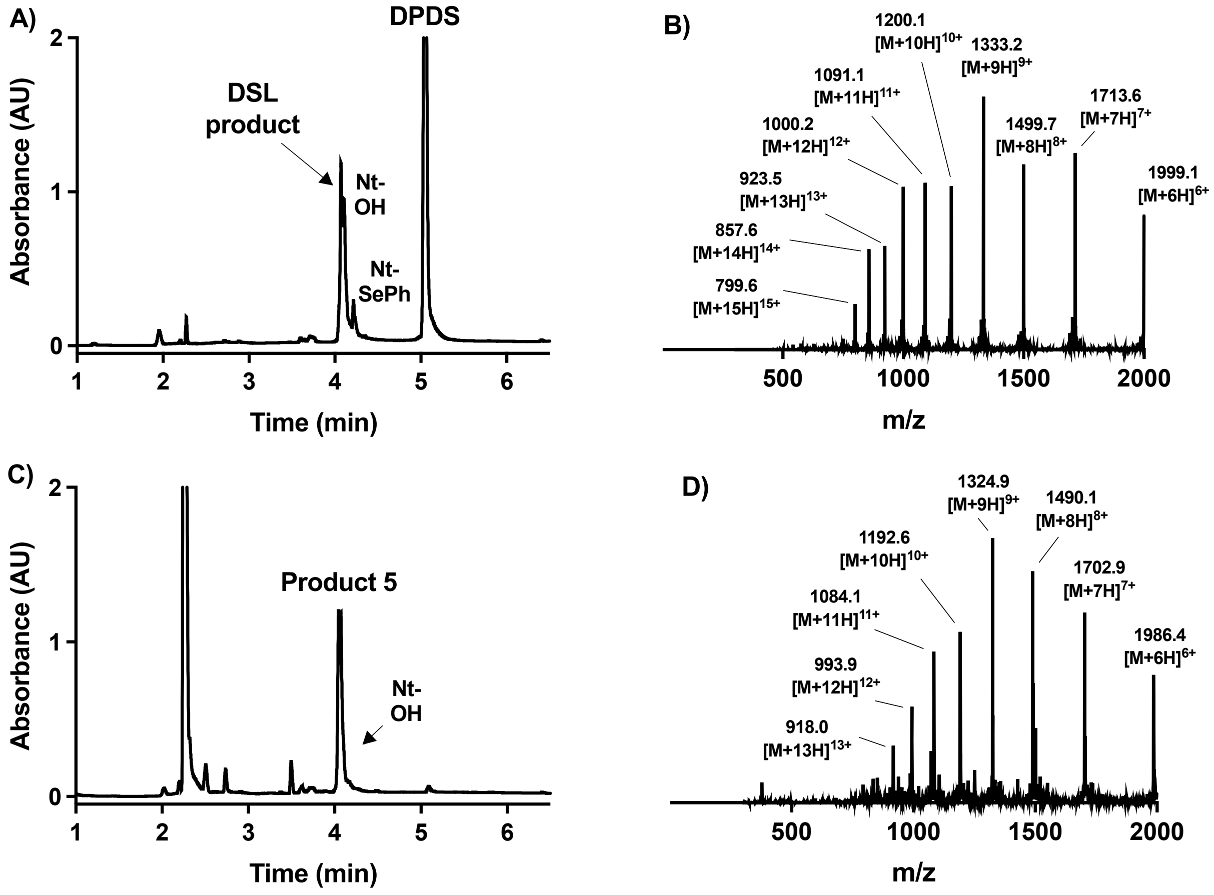

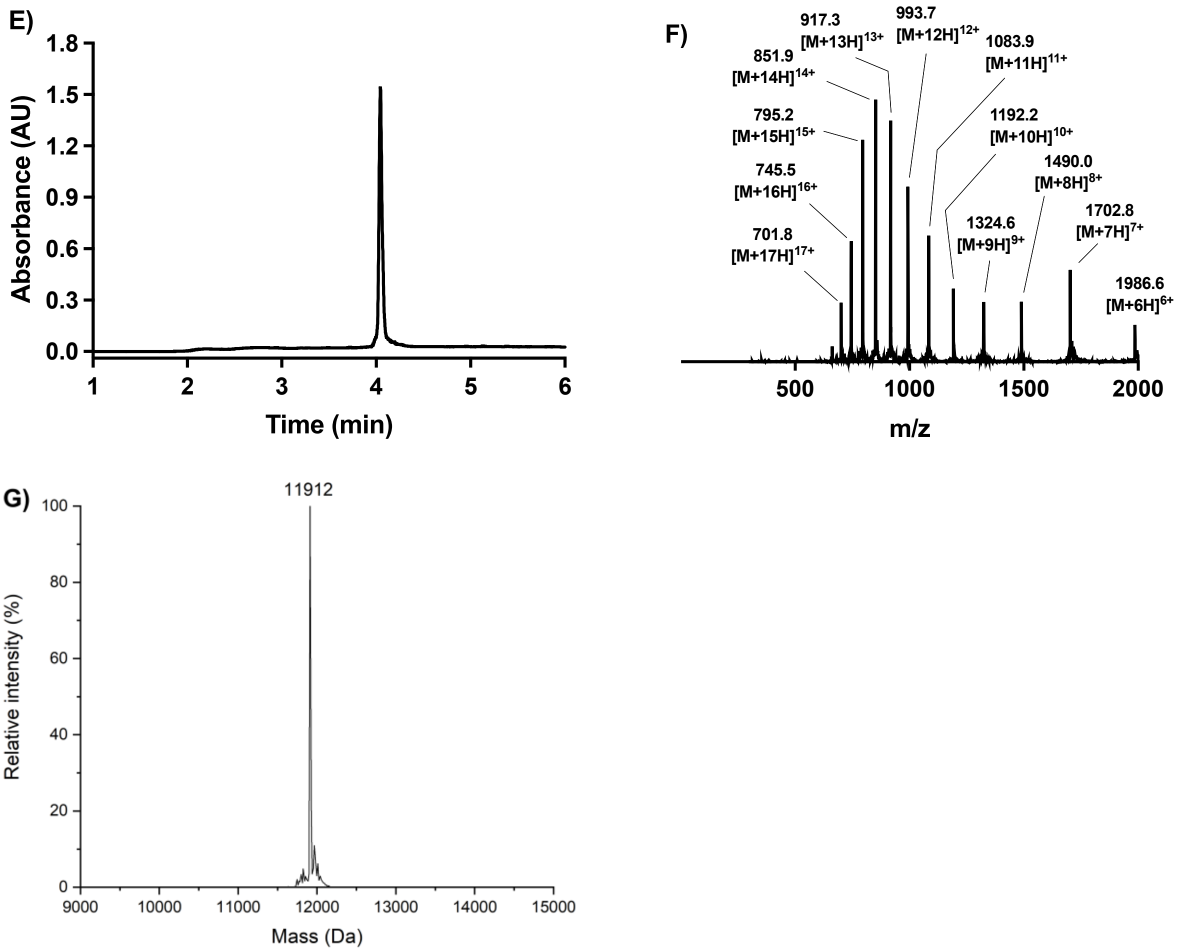

Figure S5: (A) Analytical HPLC trace of crude ligation; **(B)** ESI-MS of crude ligation; **(C)** Analytical HPLC trace of crude deselenization; **(D)** ESI-MS of crude deselenization; **(E)** Analytical HPLC trace of purified Ubiquitin-TMEM43 conjugate (**5**), R_t 4.05 min (0 to 70% B over 5 min, 0.1% v/v TFA, λ = 214 nm); **(F)** ESI-MS of pure **5**; Calculated Mass: $[M+6H]^{6+}$: 1986.6; $[M+7H]^{7+}$: 1702.9; $[M+8H]^{8+}$: 1490.2; $[M+9H]^{9+}$: 1324.7; $[M+10H]^{10+}$: 1192.4; $[M+11H]^{11+}$: 1084.0; $[M+12H]^{12+}$: 993.8; $[M+13H]^{13+}$: 917.4; $[M+14H]^{14+}$: 852.0; $[M+15H]^{15+}$: 795.2; $[M+16H]^{16+}$: 745.6; $[M+17H]^{17+}$: 701.8; Mass Found (ESI+) 1986.6 $[M+6H]^{6+}$; 1702.8 $[M+7H]^{7+}$; 1490.0 $[M+8H]^{8+}$; 1324.6 $[M+9H]^{9+}$; 1192.2 $[M+10H]^{10+}$; 1083.9 $[M+11H]^{11+}$; 993.7 $[M+12H]^{12+}$; 917.3 $[M+13H]^{13+}$; 851.9 $[M+14H]^{14+}$; 795.2 $[M+15H]^{15+}$; 745.5 $[M+16H]^{16+}$; 701.8 $[M+17H]^{17+}$. ESI-MS data in panel **(F)** was collected over the entire gradient and wash cycle of the UPLC-MS; **(G)** Deconvoluted mass spectrum of **5**.

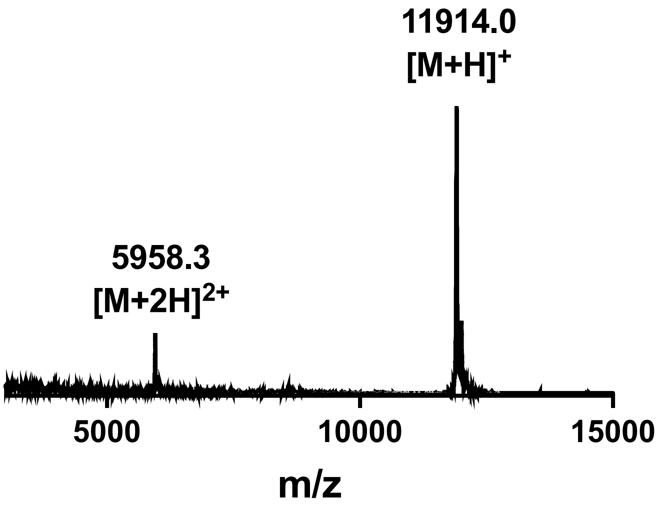

Figure S6: MALDI-TOF spectrum of Ubiquitin-TMEM43 conjugate (**5**); Calculated mass for $C_{521}H_{861}N_{150}O_{166}S^{+}$ $[M+H]^{+}$: 11914.5; $[M+2H]^{2+}$: 5957.8; Mass found: 11914.0 $[M+H]^{+}$; 5958.3 $[M+2H]^{2+}$. (Mass accuracy: Average observed mass - Average theoretical mass = -0.5 Da; -42 ppm)

Synthesis of Ubiquitin-ZAP70 conjugate:

Synthesis of ZAP70(2-26) diselenide:

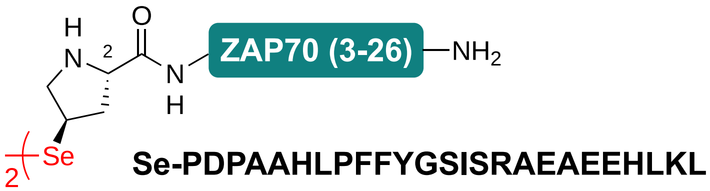

ZAP70(2-26) was prepared according to Fmoc-strategy SPPS on Rink amide resin (50 µmol) as outlined in the general procedures, using the previously reported (Boc-γ-Se-Pro-OH)$_2$ building block.[3] Removal of the acid labile protecting groups, with concurrent cleavage from resin, was achieved *via* treatment with a solution of TFA/*i*Pr$_3$SiH/H_2O (90:5:5 v/v/v, 10 mL) for 2 h at room temperature. After filtering off the resin, the deprotection solution was concentrated under nitrogen flow and the crude peptide was precipitated from ice-cold Et_2O. Purification of the crude peptide (16.7 µmol) using preparative RP-HPLC (20 to 45% B over 50 min, 0.1% v/v TFA) afforded diselenide **ZAP70(2-26)** as a fluffy white solid after lyophilization (12.6 mg, 26% yield).

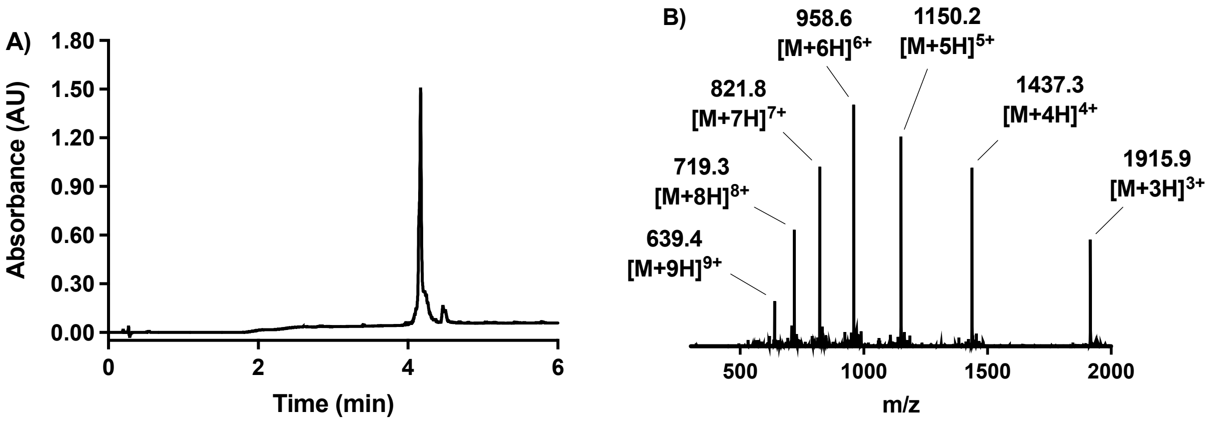

Figure S7: (A) Analytical HPLC trace of purified **ZAP70(2-26) diselenide dimer**. R_t 4.17 min (0 to 70% B over 5 min, 0.1% v/v TFA, λ = 214 nm); **(B)** ESI-MS of pure **ZAP70(2-26) diselenide dimer**; Calculated Mass: $[M+3H]^{3+}$: 1916.3; $[M+4H]^{4+}$: 1437.5; $[M+5H]^{5+}$: 1150.2; $[M+6H]^{6+}$: 958.7; $[M+7H]^{7+}$: 821.9; $[M+8H]^{8+}$: 719.3; $[M+9H]^{9+}$: 639.4. Mass Found (ESI+) 1915.9 $[M+3H]^{3+}$; 1437.3 $[M+4H]^{4+}$; 1150.2 $[M+5H]^{5+}$; 958.6 $[M+6H]^{6+}$; 821.8 $[M+7H]^{7+}$; 719.3 $[M+8H]^{8+}$; 639.4 $[M+9H]^{9+}$.

Ubiquitin-Zap70(2-26) conjugate:

Ubiquitin (1-76)-NHNH$_2$ (3.0 mg, 0.30 μmol) was subjected to the selenoesterification conditions described in the general procedure, followed by the addition of **ZAP70(2-26)** (2.0 mg, 0.62 μmol) in accordance with the general procedure for ligation-deselenization described above. Purification of the crude peptide by preparative RP-HPLC (0 to 20% B over 5 min then 20 to 50% B over 40 min at 50 °C, 0.1% v/v TFA) afforded the **Ubiquitin(1-76)-ZAP70(2-26)** conjugate as a fluffy white solid after lyophilization (0.6 mg, 15% yield, 53% yield/step over 3 steps).

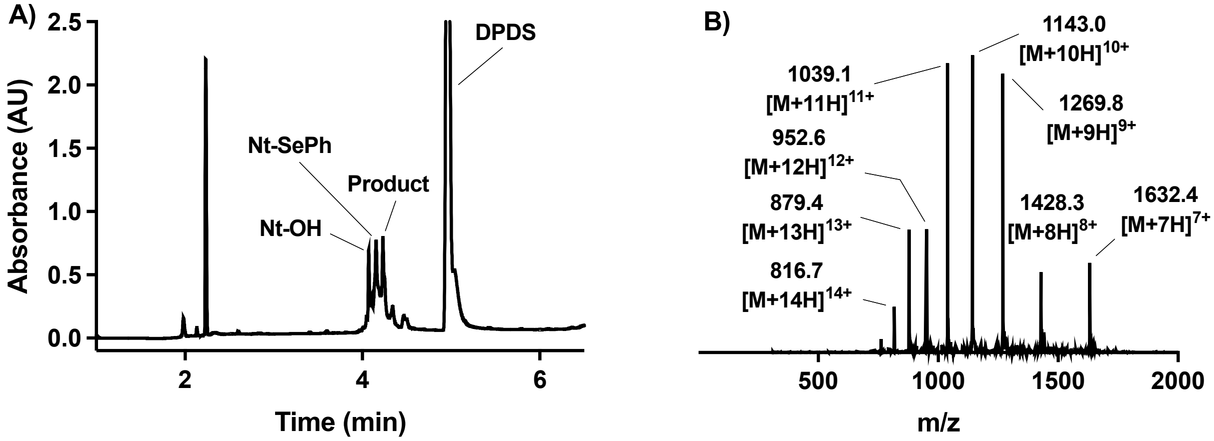

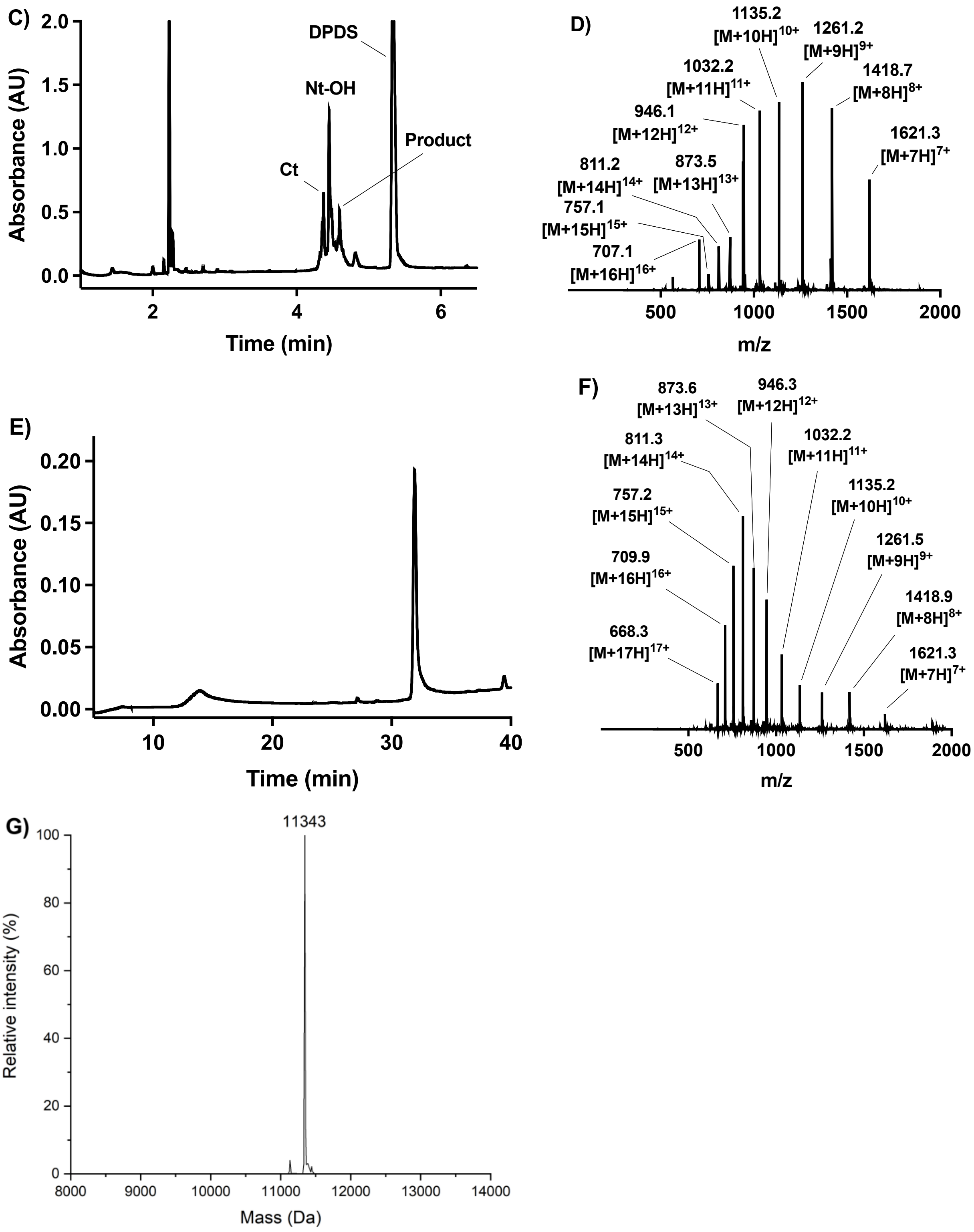

Figure S8: **(A)** Analytical HPLC trace of crude ligation; **(B)** ESI-MS of crude ligation; **(C)** Analytical HPLC trace of crude deselenization; **(D)** ESI-MS of crude deselenization; **(E)** Analytical HPLC trace of purified **Ubiquitin-ZAP70** conjugate, R_t 31.9 min (0% B for 5 min, then 0 to 60% B over 30 min, 0.1% v/v TFA, λ = 214 nm); **(F)** ESI-MS of pure **Ubiquitin-ZAP70**

conjugate; Calculated Mass: $[M+7H]^{7+}$: 1621.4; $[M+8H]^{8+}$: 1418.9; $[M+9H]^{9+}$: 1261.3; $[M+10H]^{10+}$: 1135.3; $[M+11H]^{11+}$: 1032.2; $[M+12H]^{12+}$: 946.2; $[M+13H]^{13+}$: 873.5; $[M+14H]^{14+}$: 811.2; $[M+15H]^{15+}$: 757.2; $[M+16H]^{16+}$: 709.9; $[M+17H]^{17+}$: 668.2; Mass Found (ESI+) 1621.3 $[M+7H]^{7+}$; 1418.9 $[M+8H]^{8+}$; 1261.5 $[M+9H]^{9+}$; 1135.2 $[M+10H]^{10+}$; 1032.2 $[M+11H]^{11+}$; 946.3 $[M+12H]^{12+}$; 873.6 $[M+13H]^{13+}$; 811.3 $[M+14H]^{14+}$; 757.2 $[M+15H]^{15+}$; 709.9 $[M+16H]^{16+}$; 668.3 $[M+17H]^{17+}$. ESI-MS data in panel **(F)** was collected over the entire gradient and wash cycle of the UPLC-MS; **(G)** Deconvoluted mass spectrum of **Ubiquitin-ZAP70** conjugate.

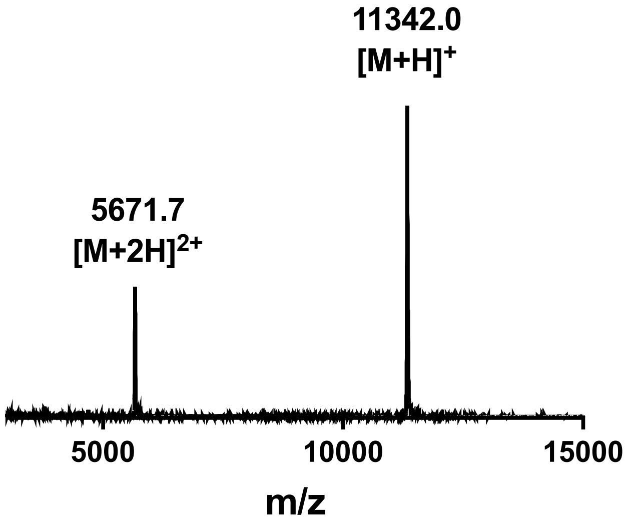

Figure S9: MALDI-TOF spectrum of **Ubiquitin-ZAP70** conjugate. Calculated Mass for $C_{507}H_{819}N_{138}O_{154}S^{+}$ $[M+H]^{+}$: 11344.0; $[M+2H]^{2+}$: 5672.5; Mass found: 11342.0 $[M+H]^{+}$; 5671.7 $[M+2H]^{2+}$. (Mass accuracy: Average observed mass - Average theoretical mass = -2 Da; -182 ppm)

Semi-synthesis of lipidated analogues of YPT6:

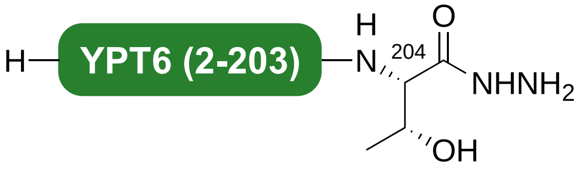

**SRSGKSLTKYKIVFLGEQGVGKTSLITRFMYDTFDDHYQATIGIDFLSKTM
YLDDKTIRLQLWDTAGQERFRSLIPSYIRDSRVAIIVYDITKRKSFEYIDKW
IEDVKNERGDENVILCIVGNKSDLSDERQISTEEGEKKAKLLGAKIFMETS
TKAGYNVKALFKKIAKSLPEFQNSESTPLDSENANSANQNKPGVIDIST**

I. Recombinant expression and purification of YPT6 (2-204)-hydrazide (8):

A synthetic YPT6 (2-204) DNA sequence (BioCat) was inserted upstream of the *Mycobacterium xenopi* DNA Gyrase A (*Mxe* GyrA) intein, a His_7 tag and a chitin-binding domain (CBD) in a pTXB1 (New England Biolabs) vector via *Nde*I and *Spe*I restriction sites. Protein expression was carried out in *E. coli* BL21(DE3) Rosetta2 (Novagen) cells in 2YT medium (16 g/L Trypton, 10 g/L Yeast extract, 5 g/L NaCl) containing 100 µg/mL ampicillin and 30 µg/mL chloramphenicol at 37 °C.
Overnight cultures were diluted to OD_{600} 0.2, grown until OD_{600} 0.7 and protein overexpression was induced with 1 mM IPTG. After 4 h, the culture was centrifuged for 20 min at 10,000 g, the cell pellets were resuspended in TBS buffer (50 mM Tris, 150 mM NaCl, pH 7.5) and lysed twice in a high-pressure cell disrupter (Constant Systems). The lysate was centrifuged for 30 min at 50,000 g. Each 45 mL supernatant was incubated with 5 mL TBS-equilibrated chitin resin (NEB) for 2 h. The resin was washed twice with 45 mL washing buffer (50 mM Tris, 500 mM NaCl) and subsequently incubated with 30 mL hydrazine solution (5% hydrazine, 50 mM Tris, 500 mM NaCl, 100 mM DTT, 0,1% Triton-X) for 2 h to cleave the intein and generate the YPT6 (2-204)-hydrazide. After the cleavage, the supernatant was separated from the chitin resin, filtered and purified by RP-HPLC on a C4 (Kromasil) column (5 to 30% B over 5 min then 30 to 75% B over 40 min, 0.1% v/v TFA, flow rate: 10 mL/min).

YPT6 (2-204) sequence without intein:

SRSGKSLTKYKIVFLGEQGVGKTSLITRFMYDTFDDHYQATIGIDFLSKTMYLDDKTIRL
QLWDTAGQERFRSLIPSYIRDSRVAIIVYDITKRKSFEYIDKWIEDVKNERGDENVILCIV
GNKSDLSDERQISTEEGEKKAKLLGAKIFMETSTKAGYNVKALFKKIAKSLPEFQNSEST
PLDSENANSANQNKPGVIDIST

***Mxe* GyrA intein:**

CITGDALVALPEGESVRIADIVPGARPNSDNAIDLKVLDRHGNPVLADRLFHSGEHPVYT
VRTVEGLRVTGTANHPLLCLVDVAGVPTLLWKLIDEIKPGDYAVIQRSAFSVDCAGFAR
GKPEFAPTTYTVGVPGLVRFLEAHHRDPDAQAIADELTDGRFYYAKVASVTDAGVQPV

YSLRVDTADHAFITNGFVSHATGLTGIHHHHHHHSGLNSGLTTNPGVSAWQVNTAYTA
GQLVTYNGKTYKCLQPHTSLAGWEPSNVPALWQLQ

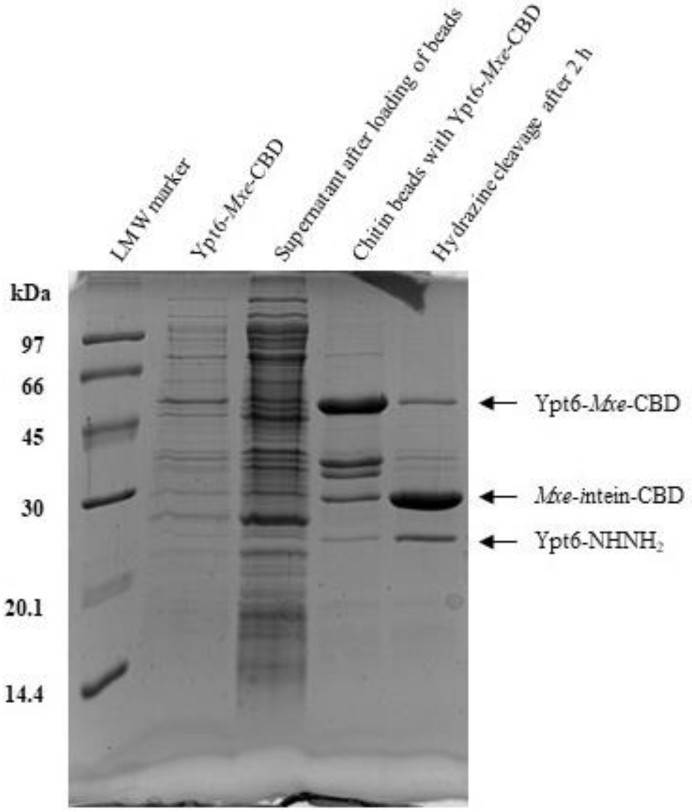

Figure S10: SDS-PAGE of YPT6(2-204) cleavage from intein fusion construct with 5% hydrazine.

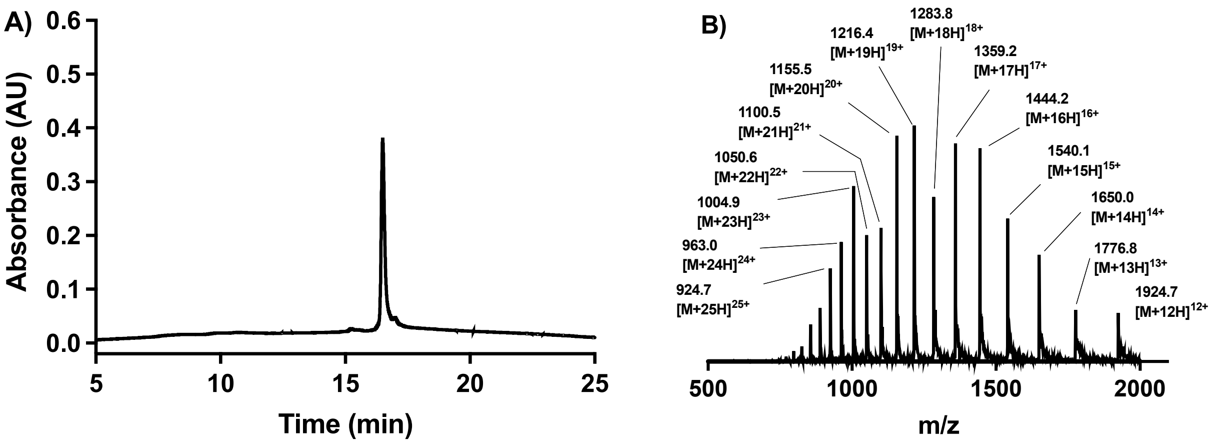

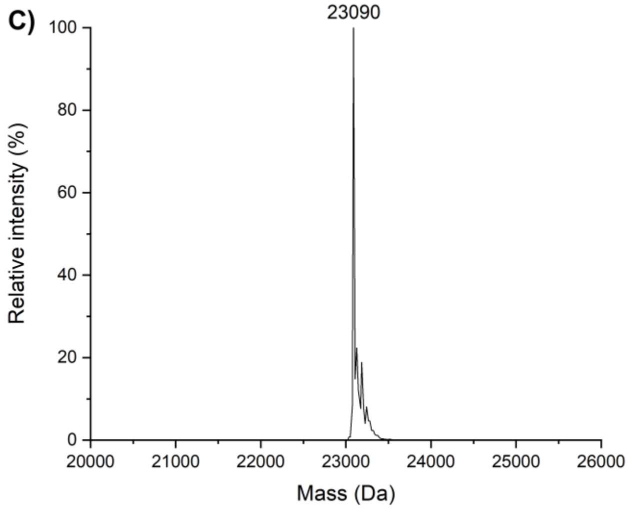

Figure S11: (A) Analytical HPLC trace of expressed YPT6-NHNH2 (**8**), R_t 16.5 min (0 to 80% B over 30 min, 0.1% v/v TFA, $\lambda = 214$ nm); (B) ESI-MS of **8**; Calculated Mass: $[M+12H]^{12+}$: 1925.0; $[M+13H]^{13+}$: 1777.0; $[M+14H]^{14+}$: 1650.1; $[M+15H]^{15+}$: 1540.2; $[M+16H]^{16+}$: 1444.0; $[M+17H]^{17+}$: 1359.1; $[M+18H]^{18+}$: 1283.7; $[M+19H]^{19+}$: 1216.2; $[M+20H]^{20+}$: 1155.4; $[M+21H]^{21+}$: 1100.4; $[M+22H]^{22+}$: 1050.5; $[M+23H]^{23+}$: 1004.8; $[M+24H]^{24+}$: 963.0; $[M+25H]^{25+}$: 924.5. Mass Found (ESI+) 1924.7 $[M+12H]^{12+}$; 1776.8 $[M+13H]^{13+}$; 1650.0 $[M+14H]^{14+}$; 1540.1 $[M+15H]^{15+}$; 1444.2 $[M+16H]^{16+}$; 1359.2 $[M+17H]^{17+}$; 1283.8 $[M+18H]^{18+}$; 1216.4 $[M+19H]^{19+}$; 1155.5 $[M+20H]^{20+}$; 1100.5 $[M+21H]^{21+}$; 1050.6 $[M+22H]^{22+}$; 1004.9 $[M+23H]^{23+}$; 963.0 $[M+24H]^{24+}$; 924.7 $[M+25H]^{25+}$. ESI-MS data was collected over the entire gradient and wash cycle of the UPLC-MS; **(C)** Deconvoluted mass spectrum of **8**.

Note: YPT6(2-204)-NHNH2 ribbon structure in the manuscript was predicted from Alphafold.[4]

II. Synthesis of YPT6 lipopeptides:

A) Synthesis of Alkylated Cysteine Building Blocks

1. (L)-2-Amino-3-dodecylsulfanyl-propionic acid methyl ester (S1)

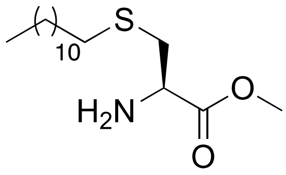

Cysteine radical alkylation was performed using methods adapted from Triola *et al.*[5] Briefly, L-cysteine methyl ester hydrochloride (1 g, 5.8 mmol) and 1-dodecene (4 mL, 17.4 mmol, 3 eq.) were dissolved in dichloroethane (25 mL) and degassed with argon. Azobisisobutyronitrile (480 mg, 2.9 mmol, 0.5 eq.) was added and the reaction was brought to reflux at 90 °C for 3 h, before the solvent was removed *in vacuo*. The crude reaction mixture was then purified *via* flash column chromatography (eluent 1:19 v/v MeOH: CH_2Cl_2) affording *S*-dodecylated Cys methyl ester **S1** as

a waxy yellow solid (1.57 g, 5.2 mmol, 89%). ^{1}H NMR (500 MHz; d^{6}-DMSO) d: 8.84 (2H, brs, N<u>H</u>$_2$), 4.20 (1H, t, J = 5.8 Hz, C<u>H</u>NH$_2$), 3.73 (3H, s, OC<u>H</u>$_3$), 3.07–3.00 (2H, m, CHC<u>H</u>$_2$), 2.53 (2H, t, J = 7.3 Hz, SC<u>H</u>$_2$CH$_2$), 1.52–1.46 (2H, m, SCH$_2$C<u>H</u>$_2$), 1.33–1.20 (18H, m, -C<u>H</u>$_2$-), 0.85 (3H, t, J = 6.8 Hz, CH$_2$C<u>H</u>$_3$). ^{13}C NMR (126 MHz; d^{6}-DMSO) d: 168.7 (C), 52.8 (CH$_3$), 52.0 (CH), 31.6 (CH$_2$), 31.3 (CH$_2$), 31.2 (CH$_2$), 29.03 (CH$_2$), 29.00 (CH$_2$), 28.99 (CH$_2$), 28.95 (CH$_2$), 28.9 (CH$_2$), 28.7 (CH$_2$), 28.6 (CH$_2$), 28.1 (CH$_2$), 22.1 (CH$_2$), 13.9 (CH$_3$).

2. (L)-2-Amino-3-hexadecylsulfanyl-propionic acid methyl ester (S2)

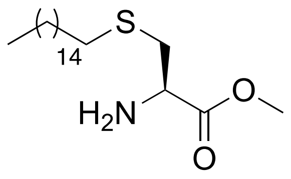

Cysteine radical alkylation was performed using methods adapted from Triola *et al.*[5] Briefly, L-cysteine methyl ester hydrochloride (1 g, 5.8 mmol) and 1-hexadecene (5.1 mL, 17.4 mmol, 3 eq.) were dissolved in dichloroethane (25 mL) and degassed with argon. Azobisisobutyronitrile (480 mg, 2.9 mmol, 0.5 eq.) was added and the reaction was brought to reflux at 90 °C for 3 h, before the solvent was removed *in vacuo*. The crude reaction mixture was then purified *via* flash column chromatography (eluent 1:9 v/v MeOH: CH$_2$Cl$_2$) affording *S*-palmitylated Cys methyl ester **S2** as a waxy yellow solid (1.75 g, 4.9 mmol, 84%). ^{1}H NMR (500 MHz; d^{6}-DMSO) d: 8.59 (2H, brs, N<u>H</u>$_2$), 4.25 (1H, t, J = 5.8 Hz, C<u>H</u>NH$_2$), 3.75 (3H, s, OC<u>H</u>$_3$), 3.02–2.97 (2H, m, CHC<u>H</u>$_2$), 2.56–2.51 (2H, m, SC<u>H</u>$_2$CH$_2$), 1.53–1.46 (2H, m, SCH$_2$C<u>H</u>$_2$), 1.34–1.19 (26H, m, -C<u>H</u>$_2$-), 0.85 (3H, t, J = 7.0 Hz, CH$_2$C<u>H</u>$_3$). ^{13}C NMR (126 MHz; d^{6}-DMSO) d: 168.8 (C), 52.8 (CH$_3$), 52.0 (CH), 31.6 (CH$_2$), 31.33 (CH$_2$), 31.26 (CH$_2$), 29.00 (4 x CH$_2$), 28.97 (3 x CH$_2$), 28.93 (CH$_2$), 28.8 (CH$_2$), 28.7 (CH$_2$), 28.6 (CH$_2$), 28.1 (CH$_2$), 22.1 (CH$_2$), 13.9 (CH$_3$).

B) YPT6 (205-215) fragments:

1. Synthesis of lipopeptide 9 (Dodecyl variant)

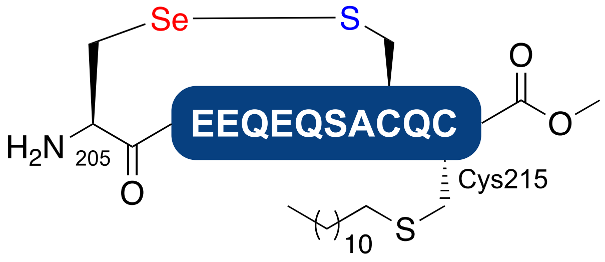

Fmoc-Glu(OH)-OAll (82 mg, 200 μmol, 2 eq.) was loaded onto Rink Amide resin (100 μmol, ~0.6 mmol/g) following the modified coupling conditions outlined in the general methods. The target peptide was extended to the penultimate residue using automated Fmoc-SPPS protocols outlined in general methods. The Fmoc-protected, resin-bound peptide was then treated with Pd(PPh$_3$)$_4$ (58 mg, 50 μmol, 0.5 eq.) and phenylsilane (247 μL, 2 mmol, 20 eq.) in CH$_2$Cl$_2$ (6 mL) to deprotect

the C-terminal allyl ester. *S*-dodecyl Cys methyl ester **S1** (61 mg, 200 µmol, 2 eq.), DIC (31 µL, 200 µmol, 2 eq.) and Oxyma (57 mg, 400 µmol, 4 eq.) in a solution of CH_2Cl_2:DMF (5 mL, v/v, 1:1) was then added to the side-chain anchored YPT6 peptide and shaken for 16 h. Following Fmoc-deprotection, the selenocysteine residue was introduced by coupling $(Boc\text{-}Sec\text{-}OH)_2$ at the N-terminus. The lipopeptide was concomitantly deprotected and liberated from resin using a cleavage cocktail consisting of TFA/*i*Pr_3SiH/H_2O (90:5:5 v/v/v, 10 mL) with the addition of NH_4I (145 mg, 1 mmol, 10 eq.) to reduce any methionine sulfoxide generated and left for 2 h at room temperature. After filtering off the resin, the deprotection solution was concentrated under nitrogen flow and the crude peptide was precipitated from ice-cold Et_2O. Purification of the crude peptide (50 µmol) by preparative RP-HPLC (5 to 90% B over 50 min, 0.1% v/v Formic acid) afforded lipopeptide **9** (dodecyl variant) featuring a selenosulfide as a fluffy white solid after lyophilization (3.1 mg, 4.2% yield).

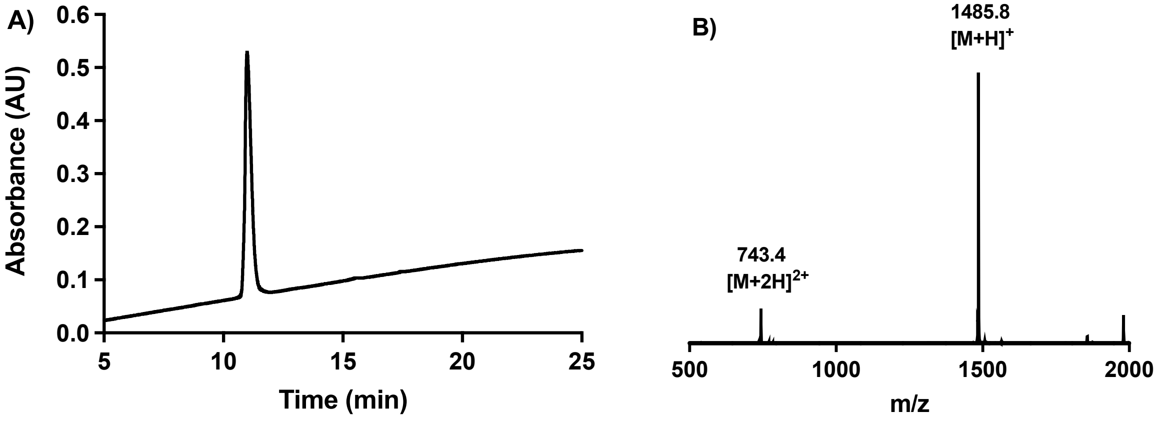

Figure S12: (A) Analytical HPLC trace of purified lipopeptide **9**, R_t 11.0 min (1 to 80% B over 30 min, 0.1% v/v TFA, λ = 214 nm); **(B)** ESI-MS of pure **9**; Calculated Mass: $[M+H]^+$: 1485.5; $[M+2H]^{2+}$: 743.3. Mass Found (ESI+) 1485.8 $[M+H]^+$; 743.4 $[M+2H]^{2+}$. ESI-MS data was collected over the entire gradient and wash cycle of the UPLC-MS.

2. Synthesis of lipopeptide 10 (Palmityl variant)

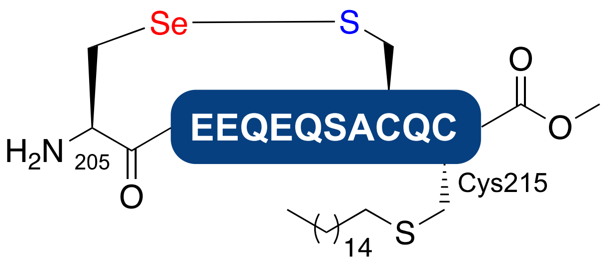

Fmoc-Glu(OH)-OAll (82 mg, 200 µmol, 2 eq.) was loaded onto Rink amide resin (100 µmol, ~0.6 mmol/g) following the modified coupling conditions outlined in the general methods. The target peptide was extended to the penultimate residue using automated Fmoc-SPPS protocols outlined in general methods. The Fmoc-protected, resin-bound peptide was then treated with $Pd(PPh_3)_4$ (58 mg, 50 µmol, 0.5 eq.) and phenylsilane (247 µL, 2 mmol, 20 eq.) in CH_2Cl_2 (6 mL) to deprotect

the C-terminal allyl ester. *S*-palmityl Cys methyl ester **S2** (72 mg, 200 µmol, 2 eq.), DIC (31 µL, 200 µmol, 2 eq.) and Oxyma (57 mg, 400 µmol, 4 eq.) in a solution of CH_2Cl_2:DMF (5 mL, v/v, 1:1) was then added to the side-chain anchored YPT6 peptide and shaken for 16 h. Following Fmoc-deprotection, the selenocysteine residue was introduced by coupling $(Boc\text{-}Sec\text{-}OH)_2$ at the N-terminus. The lipopeptide was concomitantly deprotected and liberated from resin using a cleavage cocktail consisting of TFA/*i*Pr_3SiH/H_2O (90:5:5 v/v/v, 10 mL) with the addition of NH_4I (145 mg, 1 mmol, 10 eq.) to reduce any methionine sulfoxide generated and left for 2 h at room temperature. After filtering off the resin, the deprotection solution was concentrated under nitrogen flow and the crude peptide was precipitated from ice-cold Et_2O. Purification of the crude peptide (50 µmol) by preparative RP-HPLC (3 to 90% B over 50 min, 0.1% v/v TFA) afforded lipopeptide **10** (palmityl variant) featuring a selenosulfide as a fluffy white solid after lyophilization (8.5 mg, 11% yield).

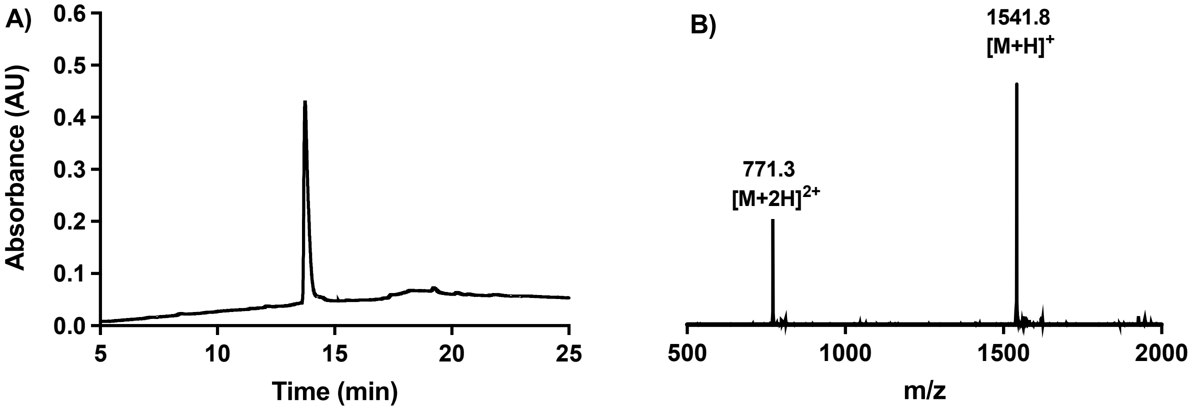

Figure S13: (A) Analytical HPLC trace of purified lipopeptide **10**, R_t 13.8 min (1 to 90% B over 30 min, 0.1% v/v TFA, λ = 214 nm); **(B)** ESI-MS of pure **10**; Calculated Mass: $[M+H]^+$: 1541.6; $[M+2H]^{2+}$: 771.3. Mass Found (ESI+) 1541.8 $[M+H]^+$; 771.3 $[M+2H]^{2+}$. ESI-MS data was collected over the entire gradient and wash cycle of the UPLC-MS.

III. Semi-synthesis of dodecylated YPT6 (6):

YPT6(2-204)-NHNH$_2$ (1.0 mg, 0.043 μmol) was subjected to the selenoester formation conditions as described in the general procedure (pH 2.0, 2.5 h, room temperature), followed by the addition of lipopeptide **9** (0.13 mg, 0.086 μmol) in accordance with the general procedure for ligation described above, with the addition of dodecylphosphocholine (DPC, 48 mM). After Et_2O extraction to remove residual DPDS, the reaction mixture was subjected to deselenization as described in the general procedure (120 mM TCEP, 120 mM reduced GSH, pH 6.5, 48 h, room temperature) followed by RP-HPLC purification using a C4 column (Symmetry C4, 300 Å, 5 μm, 2.1 mm × 150 mm; 1 to 70% B over 50 min, 0.1% v/v TFA) to afford the desired dodecylated YPT6 (**6**) (0.41 mg, 39% yield over 3 steps).

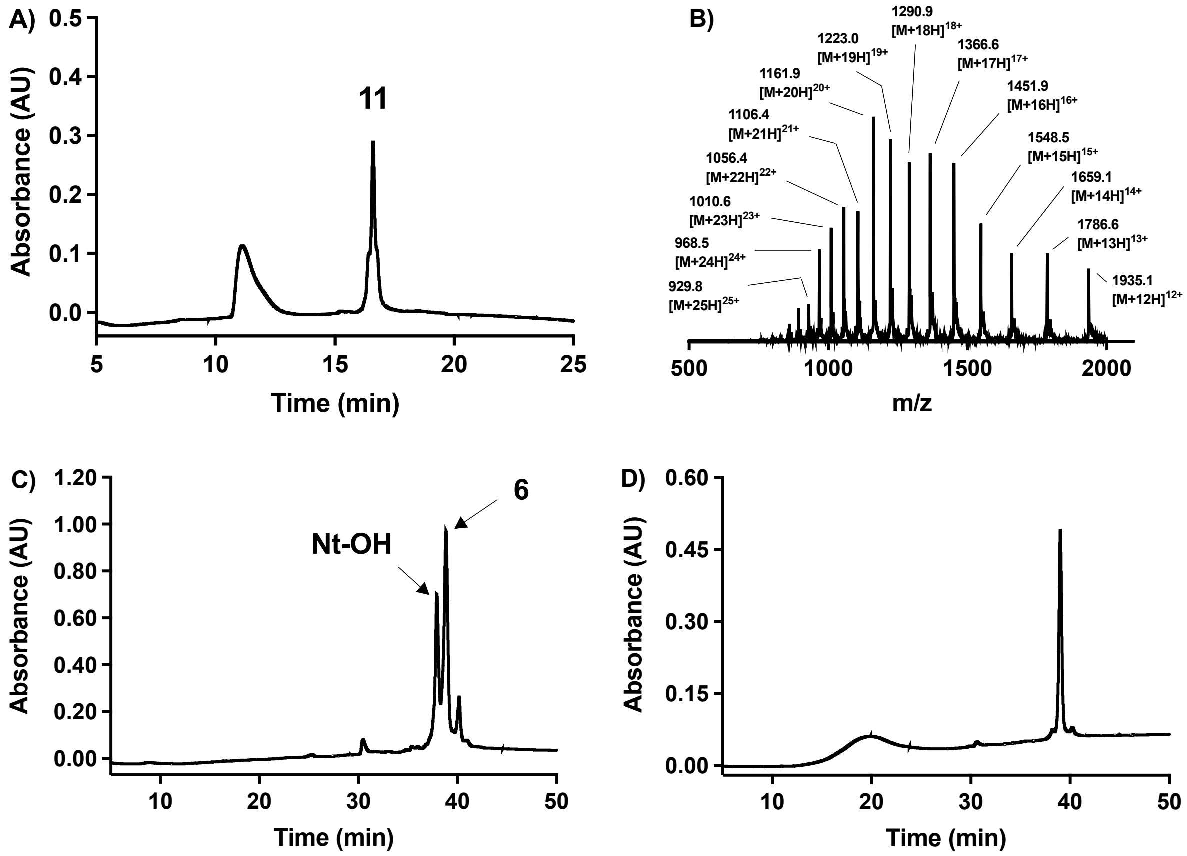

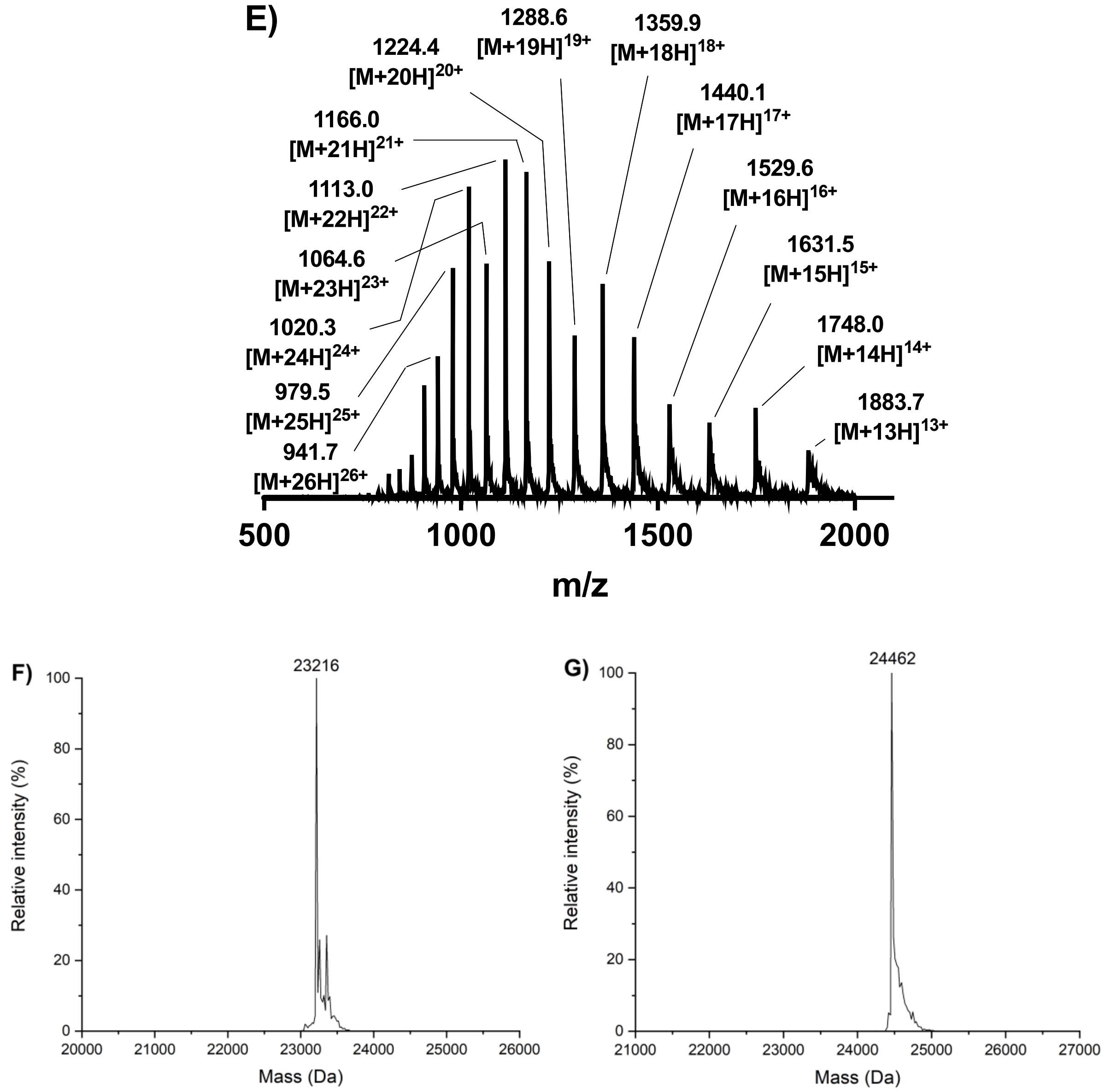

Figure S14: (A) Analytical HPLC trace of crude YPT6 selenoester **11** (0 to 80% B over 30 min, 0.1% v/v TFA, λ= 214 nm); **(B)** ESI-MS of **11**; Calculated Mass: $[M+12H]^{12+}$: 1935.4; $[M+13H]^{13+}$: 1786.6; $[M+14H]^{14+}$: 1659.1; $[M+15H]^{15+}$: 1548.5; $[M+16H]^{16+}$: 1451.8; $[M+17H]^{17+}$: 1366.5; $[M+18H]^{18+}$: 1290.6; $[M+19H]^{19+}$: 1222.7; $[M+20H]^{20+}$: 1161.7; $[M+21H]^{21+}$: 1106.4; $[M+22H]^{22+}$: 1056.1; $[M+23H]^{23+}$: 1010.3; $[M+24H]^{24+}$: 968.2; $[M+25H]^{25+}$: 929.5. Mass Found (ESI+) 1935.1 $[M+12H]^{12+}$; 1786.6 $[M+13H]^{13+}$; 1659.1 $[M+14H]^{14+}$; 1548.5 $[M+15H]^{15+}$; 1451.9 $[M+16H]^{16+}$; 1366.6 $[M+17H]^{17+}$; 1290.9 $[M+18H]^{18+}$; 1223.0 $[M+19H]^{19+}$; 1161.9 $[M+20H]^{20+}$; 1106.4 $[M+21H]^{21+}$; 1056.4 $[M+22H]^{22+}$; 1010.6 $[M+23H]^{23+}$; 968.5 $[M+24H]^{24+}$; 928.8 $[M+25H]^{25+}$. **(C)** Analytical HPLC trace of crude reaction mixture after ligation-deselenization (1 to 70% B over 50 min, 0.1% v/v TFA, λ= 214 nm); **(D)** Analytical HPLC trace of purified dodecylated YPT6 (**6**), R_t 39.0 min (1 to 70% B over 50 min, 0.1% v/v TFA, λ = 214 nm); **(E)** ESI-MS of pure **6**; Calculated Mass: $[M+13H]^{13+}$: 1882.8;

$[M+14H]^{14+}$: 1748.4; $[M+15H]^{15+}$: 1631.9; $[M+16H]^{16+}$: 1530.0; $[M+17H]^{17+}$: 1440.0; $[M+18H]^{18+}$: 1360.1; $[M+19H]^{19+}$: 1288.6; $[M+20H]^{20+}$: 1224.2; $[M+21H]^{21+}$: 1165.9; $[M+22H]^{22+}$: 1113.0; $[M+23H]^{23+}$: 1064.6; $[M+24H]^{24+}$: 1020.3; $[M+25H]^{25+}$: 979.6; $[M+26H]^{26+}$: 941.9. Mass Found (ESI+) 1883.7 $[M+13H]^{13+}$; 1748.0 $[M+14H]^{14+}$; 1631.5 $[M+15H]^{15+}$; 1529.6 $[M+16H]^{16+}$; 1440.1 $[M+17H]^{17+}$; 1359.9 $[M+18H]^{18+}$; 1288.6 $[M+19H]^{19+}$; 1224.4 $[M+20H]^{20+}$; 1166.0 $[M+21H]^{21+}$; 1113.0 $[M+22H]^{22+}$; 1064.6 $[M+23H]^{23+}$; 1020.3 $[M+24H]^{24+}$; 979.5 $[M+25H]^{25+}$; 941.7 $[M+26H]^{26+}$. ESI-MS data in panel **(E)** was collected over the entire gradient and wash cycle of the UPLC-MS; **(F)** Deconvoluted mass spectrum of **11**; **(G)** Deconvoluted mass spectrum of **6**.

IV. Semi-synthesis of palmitylated YPT6 (7):

YPT6(2-204)-NHNH$_2$ (1.0 mg, 0.043 μmol) was subjected to the selenoester formation conditions as described in the general procedure (pH 2.0, 2.5 h, room temperature), followed by the addition of palmitylated YPT6(205-215) **10** (0.13 mg, 0.086 μmol) in accordance with the general procedure for ligation described above, with the addition of dodecylphosphocholine (DPC, 48 mM). After Et_2O extraction to remove residual DPDS, the reaction mixture was subjected to deselenization as described in the general procedure (120 mM TCEP, 120 mM reduced GSH, pH 6.5, 48 h, room temperature) followed by RP-HPLC purification using a C4 column (Symmetry C4, 300 Å, 5 µm, 2.1 mm × 150 mm; 1 to 70% B over 50 min, 0.1% v/v TFA) to afford the desired palmitylated YPT6 (**7**) (0.34 mg, 32% yield over 3 steps).

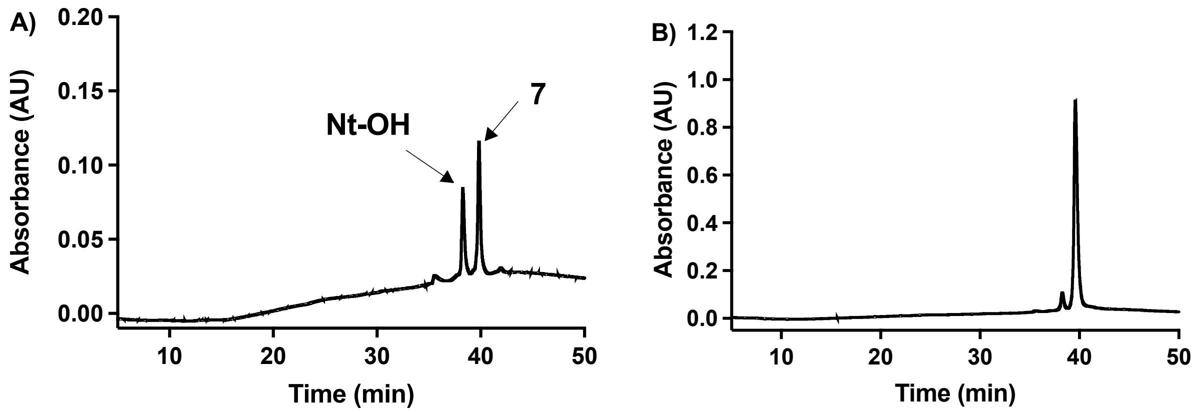

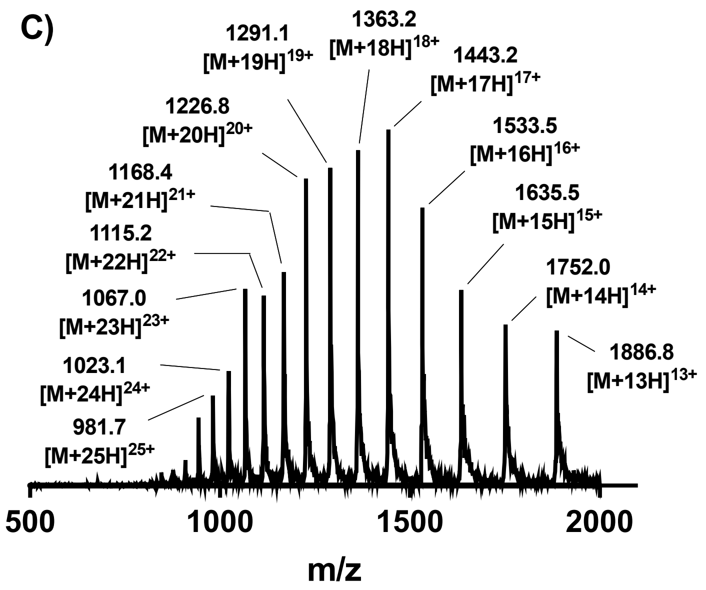

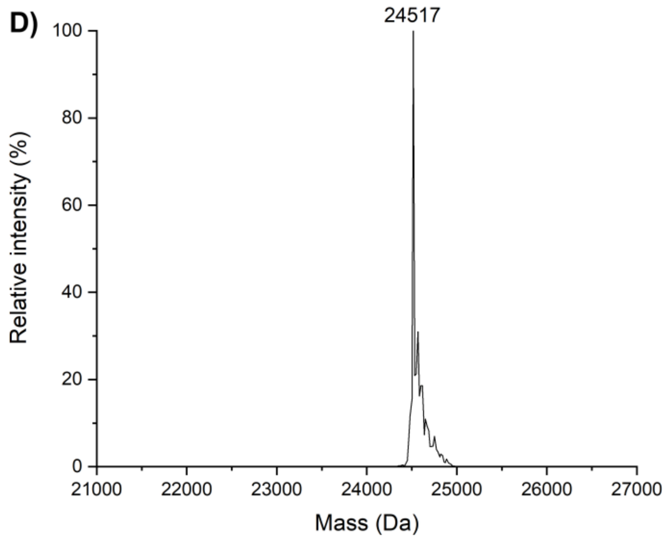

Figure S15: (A) Analytical HPLC trace of crude reaction mixture after ligation-deselenization (1 to 70% B over 50 min, 0.1% v/v TFA, λ= 214 nm); **(B)** Analytical HPLC trace of purified palmitylated YPT6 (**7**), R_t 39.6 min (1 to 70% B over 50 min, 0.1% v/v TFA, λ = 214 nm); **(C)** ESI-MS of pure **7**; Calculated Mass: $[M+13H]^{13+}$: 1887.2; $[M+14H]^{14+}$: 1752.4; $[M+15H]^{15+}$: 1635.7; $[M+16H]^{16+}$: 1533.5; $[M+17H]^{17+}$: 1443.4; $[M+18H]^{18+}$: 1363.2; $[M+19H]^{19+}$: 1291.5; $[M+20H]^{20+}$: 1227.0; $[M+21H]^{21+}$: 1168.6; $[M+22H]^{22+}$: 1115.5; $[M+23H]^{23+}$: 1067.1; $[M+24H]^{24+}$: 1022.7; $[M+25H]^{25+}$: 981.8. Mass Found (ESI+) 1886.8 $[M+13H]^{13+}$; 1752.0 $[M+14H]^{14+}$; 1635.5 $[M+15H]^{15+}$; 1533.5 $[M+16H]^{16+}$; 1443.2 $[M+17H]^{17+}$; 1363.2 $[M+18H]^{18+}$; 1291.1 $[M+19H]^{19+}$; 1226.8 $[M+20H]^{20+}$; 1168.4 $[M+21H]^{21+}$; 1115.2 $[M+22H]^{22+}$; 1067.0 $[M+23H]^{23+}$; 1023.1 $[M+24H]^{24+}$; 981.7 $[M+25H]^{25+}$. ESI-MS data was collected over the entire gradient and wash cycle of the UPLC-MS; **(D)** Deconvoluted mass spectrum of **7**.

Folding of semisynthetic lipidated analogues of YPT6:

Lipidated YPT6 variants **6** and **7** were dissolved in folding buffer (50 mM Tris.HCl, 25 mM NaCl, 1 mM TCEP, 2 mM $MgCl_2$, 10 µM GTP, pH 7) containing 6 M Gnd.HCl and 1% dodecylmaltoside (DDM) to reach a final concentration of 50 µM for dodecylated YPT6 (**6**) and 10 µM of palmitylated YPT6 (**7**). The protein solutions were dialyzed stepwise against 100 volumes of the same buffer containing 3 M Gnd.HCl and subsequently against this buffer without Gnd.HCl and TCEP and finally against buffer also without GTP. Each dialysis step was performed at 4°C for 10-16 h.

After centrifugation, the supernatant was concentrated in an Amicon Ultra-0.5 device with a 10 kDa molecular weight cut-off and analyzed via RP-HPLC by isocratic elution with 50 mM potassium phosphate buffer at pH 7 containing 10 mM tetrabutylammonium bromide (TBAB) and 8% MeCN. Absorption of nucleotides was followed at 256 nm.

For the folding of dodecylated YPT6 (**6**) at 50 µM initial concentration, no signal for nucleotide bound to the protein after dialysis and concentration was detected. However, analysis of the precipitate collected after dialysis by SDS-PAGE revealed that dodecylated YPT6 (**6**) was precipitated (Figure S16). Higher molecular weight species also indicate irreversible aggregation.

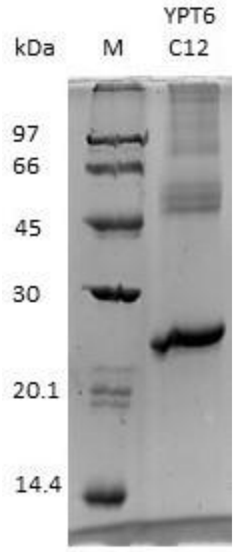

Figure S16: SDS-PAGE of precipitated dodecylated YPT6 variant **6** (YPT6 C12) after folding dissolved in sample buffer containing SDS and β-mercaptoethanol.

Palmitylated YPT6 (**7**) was dissolved at 10 µM initial concentration and no precipitated protein was detected after folding and centrifugation. Analysis of the soluble protein revealed a mixture

of GTP and GDP bound to the protein, indicating folding (Figure S17). The ratio of GDP to GTP is increased due to the intrinsic, low GTPase activity of YPT6.

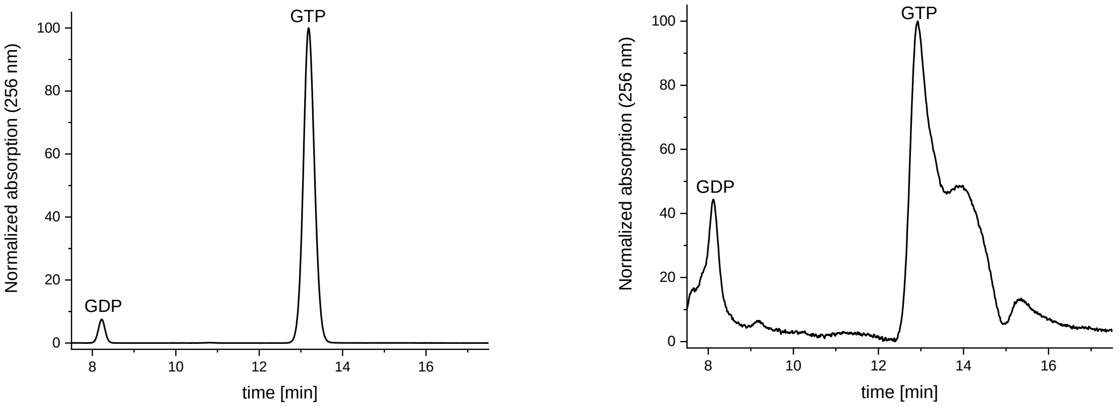

Figure S17: RP-HPLC analysis of nucleotides. **Left**: GTP used in this experiment. **Right**: Palmitylated YPT6 (**7**) after folding showing GTP and GDP bound to the protein.

Semi-synthesis of Hsp27 phosphoforms:

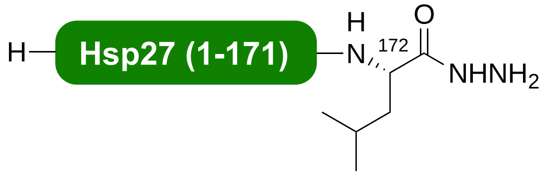

**MTERRVPFSLLRGPSWDPFRDWYPHSRLFDQAFGLPRLPEEWSQWLG
GSSWPGYVRPLPPAAIESPAVAAPAYSRALSRQLSSGVSEIRHTADRWR
VSLDVNHFAPDELTVKTKDGVVEITGKHEERQDEHGYISRCFTRKYTLPP
GVDPTQVSSSLSPEGTLTVEAPMPKL**

Recombinant Expression and Purification of Hsp27(1-172) hydrazide (15):

The Hsp27(1-172)-intein fusion protein was expressed from an already available plasmid pTXB3-Hsp27-Mxe-CBD containing the human Hsp27(1-172) DNA sequence followed by the *Mxe* GyrA intein, a His_7 tag and a chitin binding domain.[6] Protein expression was performed in *E. coli* BL21(DE3) Rosetta 2 grown in 2YT medium (16 g/L Trypton, 10 g/L Yeast extract, 5 g/L NaCl) containing 100 µg/mL ampicillin and 30 µg/mL chloramphenicol at 37 °C.

Overnight cultures were diluted to OD_{600} 0.2, grown until OD_{600} 0.7 and protein overexpression was induced by addition of 1 mM IPTG. After 4 h, the culture was centrifuged for 20 min at 10,000 g, the cell pellets were resuspended in TBS buffer (50 mM Tris, 150 mM NaCl, pH 7.5) and lysed twice in a high-pressure cell disrupter (Constant Systems). The lysate was centrifuged for 30 min at 50,000 g to separate soluble and insoluble fractions. The overexpressed Hsp27 intein fusion construct was isolated from the insoluble fraction (inclusion body) by dissolving the pellet obtained from 2 L culture in 25 mL of solubilization buffer (6 Gnd·HCl, 50 mM Tris, pH 7.5). After another centrifugation at 50,000 g the clear protein solution was transferred into a Sephadex G-25 PD10 column (Cytiva) and subjected to a buffer exchange into urea buffer (8 M urea in 50 mM Tris.HCl, pH 7.5). Intein cleavage was initiated by diluting the buffer exchanged protein solution 1:1 with 10% hydrazine, 200 mM DTT (final concentration 5% hydrazine, 100 mM DTT, 4 M urea) and incubating at room temperature for 24 h. After cleavage, the Hsp27(1-172) hydrazide **15** was purified by RP-HPLC on a C4 (Kromasil) column (25 to 70% B over 40 min, 0.1% v/v TFA, flow rate: 10 mL/min).

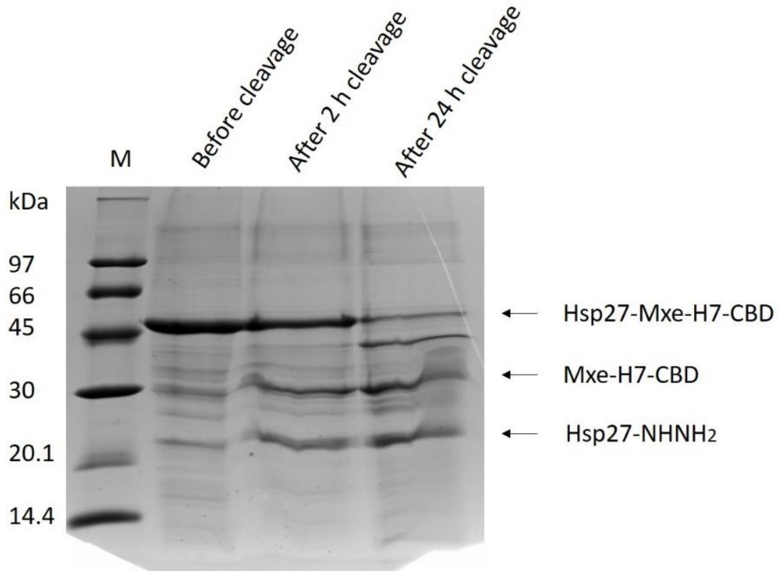

Figure S18: SDS-PAGE of Hsp27(1-172) cleavage from intein fusion construct in buffer containing 4 M urea with 5% hydrazine.

Hsp27(1-172) sequence without intein:

MTERRVPFSL LRGPSWDPFR DWYPHSRLFD QAFGLPRLPE EWSQWLGGSS WPGYVRPLPP AAIESPAVAA PAYSRALSRQ LSSGVSEIRH TADRWRVSLD VNHFAPDELT VKTKDGVVEI TGKHEERQDE HGYISRCFTR KYTLPPGVDP TQVSSSLSPE GTLTVEAPMP KL

Mxe GyrA intein:

CITGDALVALPEGESVRIADIVPGARPNSDNAIDLKVLDRHGNPVLADRLFHSGEHPVYT VRTVEGLRVTGTANHPLLCLVDVAGVPTLLWKLIDEIKPGDYAVIQRSAFSVDCAGFAR GKPEFAPTTYTVGVPGLVRFLEAHHRDPDAQAIADELTDGRFYYAKVASVTDAGVQPV YSLRVDTADHAFITNGFVSHATGLTGIHHHHHHHSGLNSGLTTNPGVSAWQVNTAYTA GQLVTYNGKTYKCLQPHTSLAGWEPSNVPALWQLQ

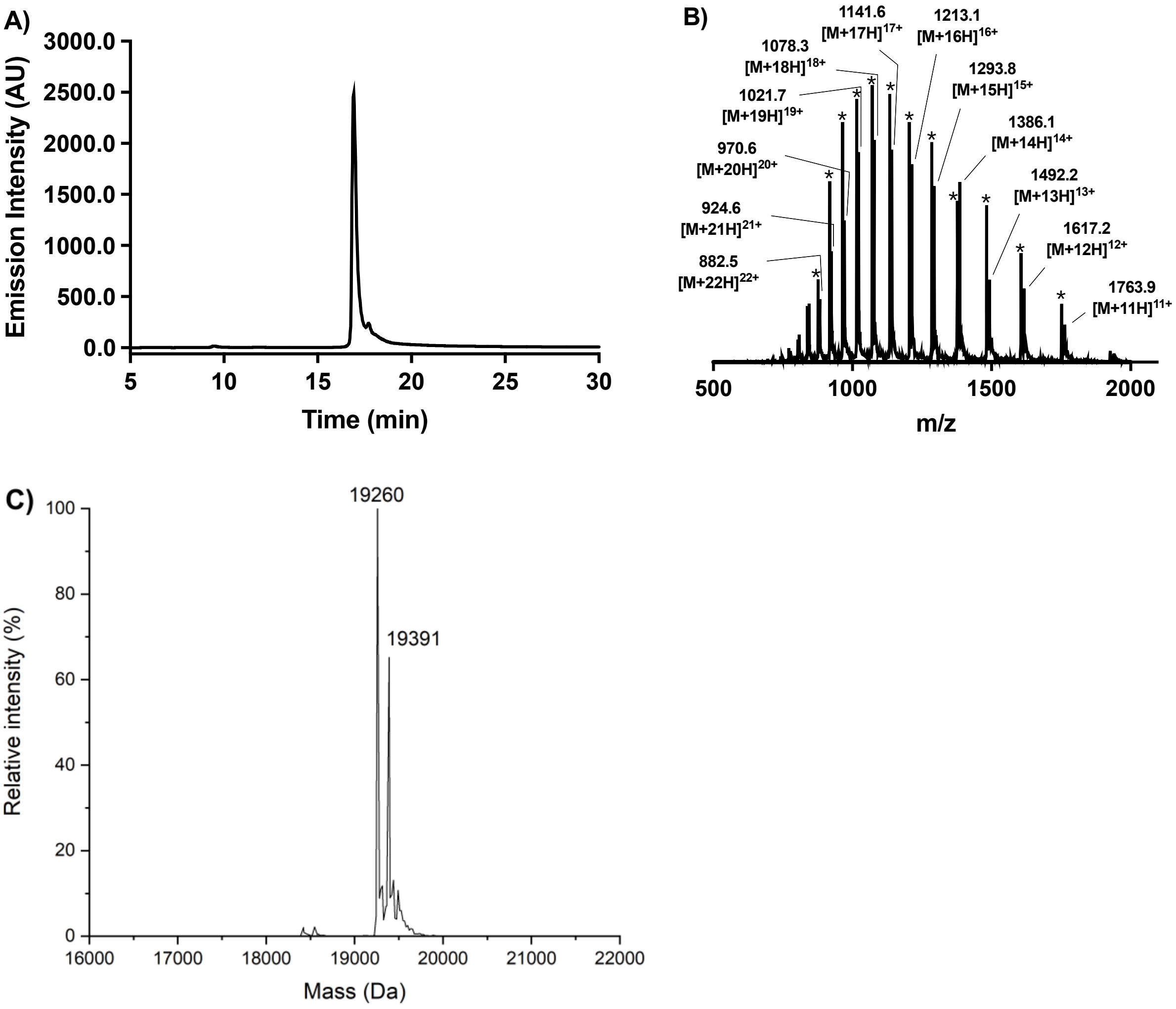

Figure S19: (A) Analytical HPLC FLR trace of **Hsp27 (1-172)-NHNH$_2$** (**15**), R_t 16.9 min (0 to 70% B over 30 min, 0.1% v/v TFA, λ_{ex} = 280 nm, λ_{em} = 347 nm); **(B)** ESI-MS of pure **15**; Calculated Mass: $[M+11H]^{11+}$: 1764.3; $[M+12H]^{12+}$: 1617.3; $[M+13H]^{13+}$: 1493.0; $[M+14H]^{14+}$: 1386.4; $[M+15H]^{15+}$: 1294.1; $[M+16H]^{16+}$: 1213.2; $[M+17H]^{17+}$: 1141.9; $[M+18H]^{18+}$: 1078.5; $[M+19H]^{19+}$: 1021.8; $[M+20H]^{20+}$: 970.8; $[M+21H]^{21+}$: 924.6; $[M+22H]^{22+}$: 882.6. Mass Found (ESI+) 1763.9 $[M+11H]^{11+}$; 1617.2 $[M+12H]^{12+}$; 1492.2 $[M+13H]^{13+}$; 1386.1 $[M+14H]^{14+}$; 1293.8 $[M+15H]^{15+}$; 1213.1 $[M+16H]^{16+}$; 1141.6 $[M+17H]^{17+}$; 1078.3 $[M+18H]^{18+}$; 1021.7 $[M+19H]^{19+}$; 970.6 $[M+20H]^{20+}$; 924.6 $[M+21H]^{21+}$; 882.5 $[M+22H]^{22+}$. ESI-MS data was collected over the entire gradient and wash cycle of the UPLC-MS; [***** = Masses correspond to partial removal of the N-terminal methionine (MW_{Met} = 131 Da) in the expressed Hsp27 (1-172)-NHNH$_2$. This is often observed for proteins overexpressed in bacteria and has no functional consequences].[6] **(C)** Deconvoluted mass spectrum of **15**; The masses correspond to the presence and absence of the N-terminal methionine derived from the expressed Hsp27 segment (*vide supra*).

Note: Hsp27(1-172)-NHNH$_2$ ribbon structure in the manuscript was predicted from Alphafold.[4]

B) Synthesis of peptide fragments:

1. Hsp27 (173-205, *p*Thr174) diselenide (17):

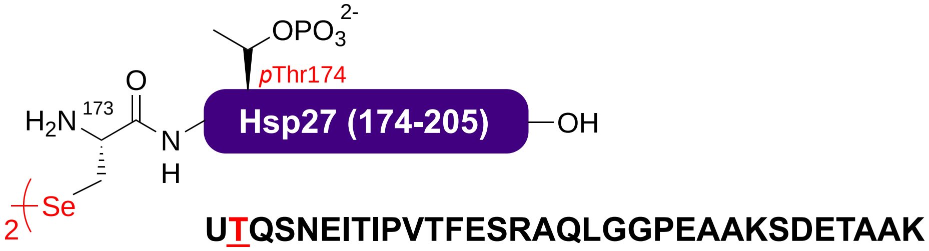

The phosphorylated selenopeptide fragment was prepared according to Fmoc-strategy SPPS on Wang resin (25 µmol) with an N-terminal Boc-Sec(PMB)-OH as outlined in the general procedures. Fmoc-Thr(PO(OBzl)OH)-OH and Boc-Sec(PMB)-OH building blocks were coupled using HATU coupling conditions [amino acid (2.5 eq.), HATU (2.5 eq.), Oxyma (2.5 eq.) and DIPEA (7.5 eq.) in DMF (0.1 M) for 16 h at room temperature]. Removal of the acid labile protecting groups, with concomitant cleavage from resin, was achieved *via* treatment with a solution of TFA/iPr$_3$SiH/H$_2$O (90:5:5 v/v/v, 3 mL) for 2 h at room temperature. After filtering off the resin, the deprotection solution was concentrated under nitrogen flow and the crude peptide was precipitated from ice-cold Et$_2$O. The crude peptide was purified, subjected to PMB deprotection (using standard conditions outlined in the general procedures) and purified by RP-HPLC (0 to 60% B over 55 min, 0.1% v/v TFA) to afford Hsp27 (*p*Thr174) diselenide **17** as a fluffy white solid after lyophilization (2.0 mg, 2.2% yield).

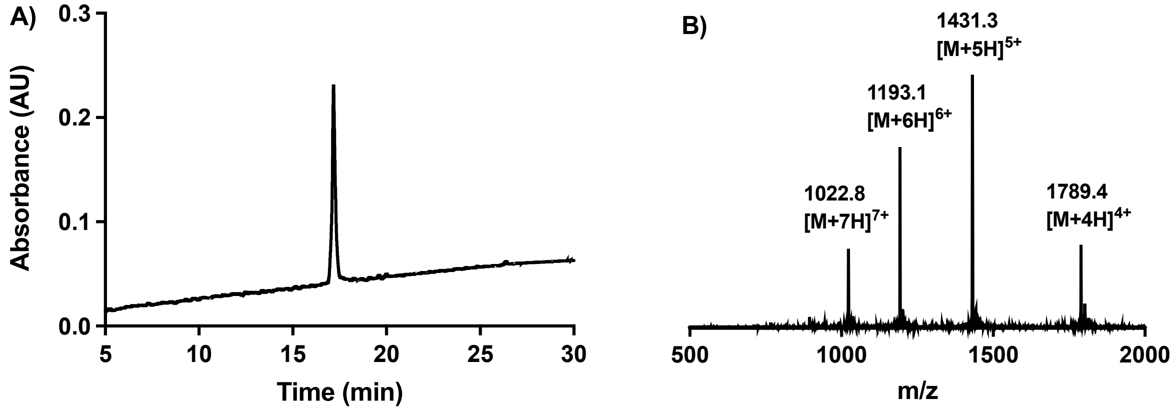

Figure S20: (A) Analytical HPLC trace of purified Hsp27 (*p*Thr174) diselenide dimer **17**, R$_t$ 17.2 min (0 to 60% B over 30 min, 0.1% v/v TFA, λ = 214 nm); **(B)** ESI-MS of pure **17**; Calculated Mass: $[M+4H]^{4+}$: 1789.3; $[M+5H]^{5+}$: 1431.7; $[M+6H]^{6+}$: 1193.2; $[M+7H]^{7+}$: 1022.9. Mass Found (ESI+) 1789.4 $[M+4H]^{4+}$; 1431.3 $[M+5H]^{5+}$; 1193.1 $[M+6H]^{6+}$; 1022.8 $[M+7H]^{7+}$. ESI-MS data was collected over the entire gradient and wash cycle of the UPLC-MS.

2. Hsp27 (173-205, *p*Ser176) diselenide (18):

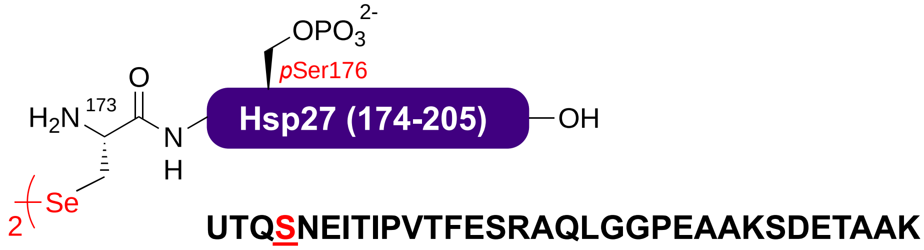

The phosphorylated selenopeptide fragment was prepared according to Fmoc-strategy SPPS on Wang resin (25 µmol) with an N-terminal Boc-Sec(PMB)-OH as outlined in the general procedures. Fmoc-Ser(PO(OBzl)OH)-OH and subsequent N-terminal residues including Boc-Sec(PMB)-OH were coupled using HATU coupling conditions [amino acid (2.5 eq.), HATU (2.5 eq.), Oxyma (2.5 eq.) and DIPEA (7.5 eq.) in DMF (0.1 M) for 16 h at room temperature]. Removal of the acid labile protecting groups, with concomitant cleavage from resin, was achieved *via* treatment with a solution of TFA/*i*Pr_3SiH/H_2O (90:5:5 v/v/v, 3 mL) for 2 h at room temperature. After filtering off the resin, the deprotection solution was concentrated under nitrogen flow and the crude peptide was precipitated from ice-cold Et_2O. The crude peptide was purified, subjected to PMB deprotection (using standard conditions outlined in the general procedures) and purified by RP-HPLC (0 to 60% B over 55 min, 0.1% v/v TFA) to afford Hsp27 (*p*Ser176) diselenide **18** as a fluffy white solid after lyophilization (6.0 mg, 7% yield).

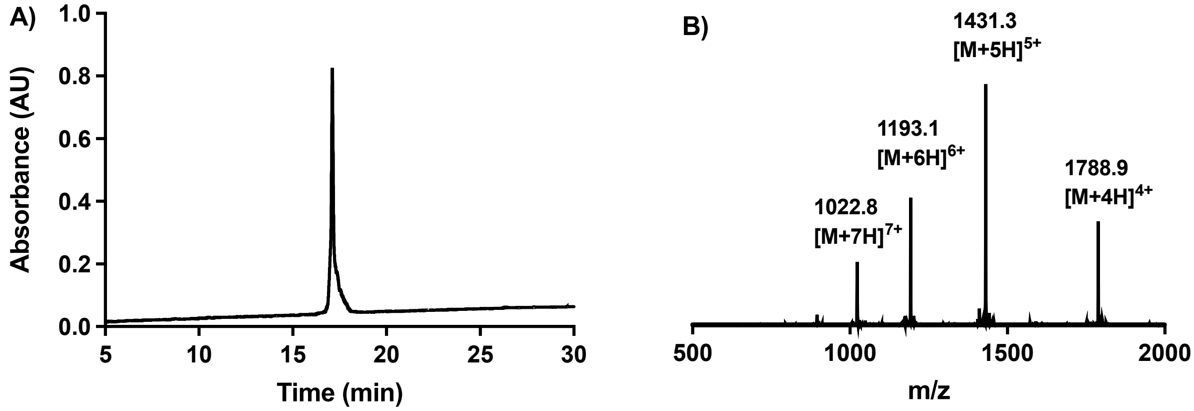

Figure S21: (A) Analytical HPLC trace of purified Hsp27 (*p*Ser176) diselenide dimer **18**, R_t 17.1 min (0 to 60% B over 30 min, 0.1% v/v TFA, λ = 214 nm); **(B)** ESI-MS of pure **18**; Calculated Mass: $[M+4H]^{4+}$: 1789.3; $[M+5H]^{5+}$: 1431.7; $[M+6H]^{6+}$: 1193.2; $[M+7H]^{7+}$: 1022.9. Mass Found (ESI+) 1788.9 $[M+4H]^{4+}$; 1431.3 $[M+5H]^{5+}$; 1193.1 $[M+6H]^{6+}$; 1022.8 $[M+7H]^{7+}$. ESI-MS data was collected over the entire gradient and wash cycle of the UPLC-MS.

3. Hsp27 (173-205, *p*Ser199) diselenide (19):

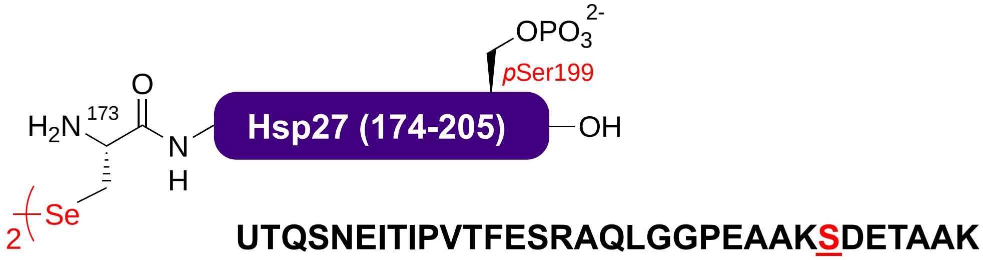

The phosphorylated selenopeptide fragment was prepared according to Fmoc-strategy SPPS on Wang resin (25 µmol) with an N-terminal Boc-Sec(PMB)-OH as outlined in the general procedures. Fmoc-Ser(PO(OBzl)OH)-OH and subsequent N-terminal residues including Boc-Sec(PMB)-OH were coupled using HATU coupling conditions [amino acid (2.5 eq.), HATU (2.5 eq.), Oxyma (2.5 eq.) and DIPEA (7.5 eq.) in DMF (0.1 M) for 16 h at room temperature]. Removal of the acid labile protecting groups, with concomitant cleavage from resin, was achieved *via* treatment with a solution of TFA/*i*Pr_3SiH/H_2O (90:5:5 v/v/v, 3 mL) for 2 h at room temperature. After filtering off the resin, the deprotection solution was concentrated under nitrogen flow and the crude peptide was precipitated from ice-cold Et_2O. The crude peptide was purified, subjected to PMB deprotection (using standard conditions outlined in the general procedures) and purified by RP-HPLC (0 to 60% B over 55 min, 0.1% v/v TFA) to afford Hsp27 (*p*Ser199) diselenide **19** as a fluffy white solid after lyophilization (4.0 mg, 4.5% yield).

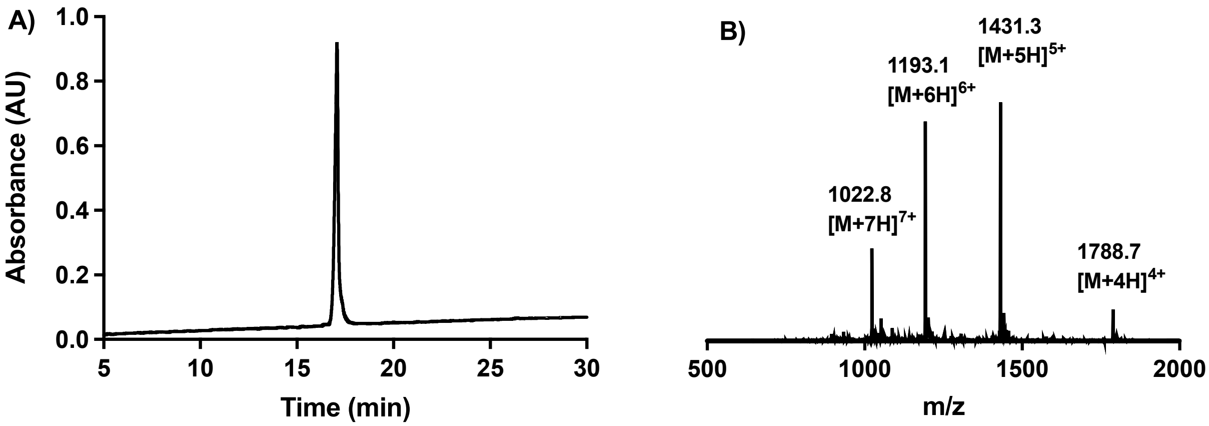

Figure S22: (A) Analytical HPLC trace of purified Hsp27 (*p*Ser199) diselenide dimer **19**, R_t 17.1 min (0 to 60% B over 30 min, 0.1% v/v TFA, λ = 214 nm); **(B)** ESI-MS of pure **19**; Calculated Mass: [M+4H]$^{4+}$: 1789.3; [M+5H]$^{5+}$: 1431.7; [M+6H]$^{6+}$: 1193.2; [M+7H]$^{7+}$: 1022.9. Mass Found (ESI+) 1788.7 [M+4H]$^{4+}$; 1431.3 [M+5H]$^{5+}$; 1193.1 [M+6H]$^{6+}$; 1022.8 [M+7H]$^{7+}$. ESI-MS data was collected over the entire gradient and wash cycle of the UPLC-MS.

C) Semi-synthesis of Hsp27 phosphoforms via one-pot selenoesterification, ligation and deselenization:

1. Hsp27 (*p*Thr174) (12):

Hsp27 (*p*Thr174) (12) was synthesized by subjecting **Hsp27 (1-172)-NHNH$_2$ 15** (2.0 mg, 0.103 μmol) to the selenoesterification conditions described in the general procedure, followed by the addition of the **Hsp27 (173-205, *p*Thr174) diselenide 17** (0.74 mg, 0.206 μmol) in accordance with the general procedure for ligation described above. After Et$_2$O extraction to remove residual DPDS, the reaction mixture was subjected to deselenization as described in the general procedure (60 mM TCEP, 60 mM reduced GSH, pH 6.5, 16 h, room temperature) to afford full length **Hsp27 (*p*Thr174)** (**12**) as a fluffy white solid after RP-HPLC purification (0 to 15% B over 5 min then 15 to 75% B over 70 min, 0.1% v/v TFA) and lyophilization (0.95 mg, 40% yield over 3 steps).

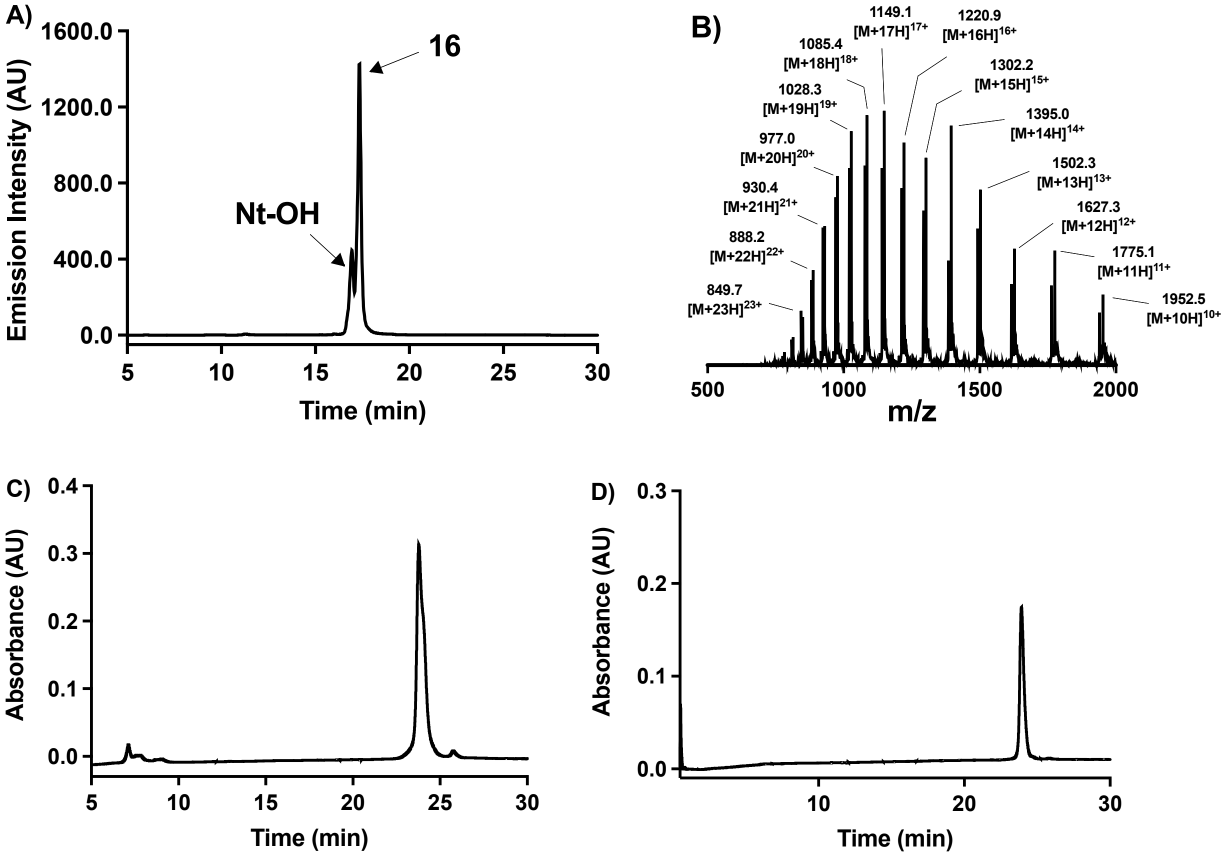

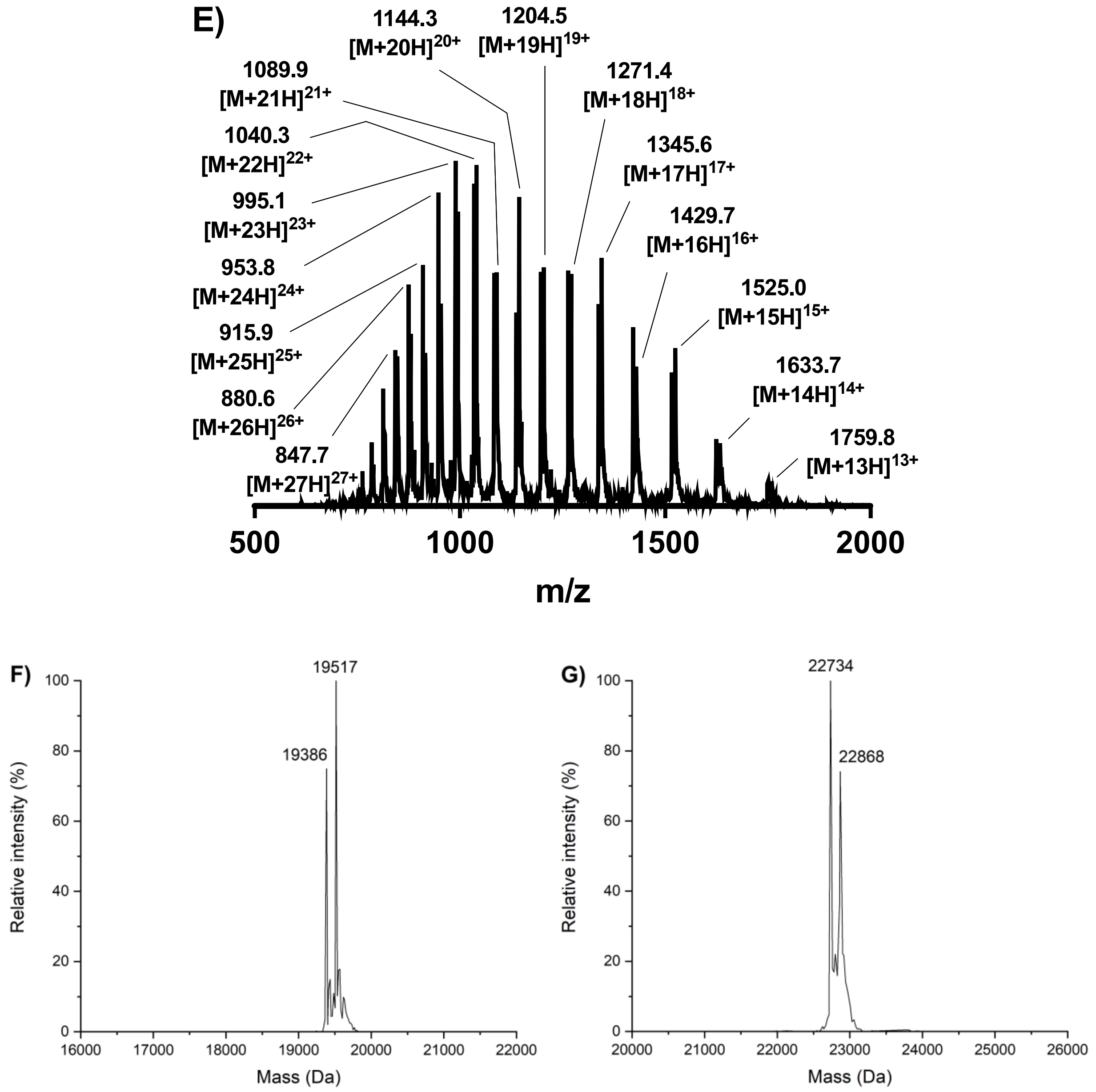

Figure S23: (A) Analytical HPLC FLR trace of crude **Hsp27 (1-172)-SePh** (**16**), R_t 17.4 min (0 to 70% B over 30 min, 0.1% v/v TFA, λ_{ex} = 280 nm, λ_{em} = 347 nm); **(B)** ESI-MS of crude **16**; Calculated Mass: $[M+10H]^{10+}$: 1953.1; $[M+11H]^{11+}$: 1775.6; $[M+12H]^{12+}$: 1627.7; $[M+13H]^{13+}$: 1502.6; $[M+14H]^{14+}$: 1395.3; $[M+15H]^{15+}$: 1302.4; $[M+16H]^{16+}$: 1221.1; $[M+17H]^{17+}$: 1149.3; $[M+18H]^{18+}$: 1085.5; $[M+19H]^{19+}$: 1028.4; $[M+20H]^{20+}$: 977.0; $[M+21H]^{21+}$: 930.6; $[M+22H]^{22+}$: 888.3; $[M+23H]^{23+}$: 849.7. Mass Found (ESI+) 1952.5 $[M+10H]^{10+}$; 1775.1 $[M+11H]^{11+}$; 1627.3 $[M+12H]^{12+}$; 1502.3 $[M+13H]^{13+}$; 1395.0 $[M+14H]^{14+}$; 1302.2 $[M+15H]^{15+}$; 1220.9 $[M+16H]^{16+}$; 1149.1 $[M+17H]^{17+}$; 1085.4 $[M+18H]^{18+}$; 1028.3 $[M+19H]^{19+}$; 977.0 $[M+20H]^{20+}$; 930.4 $[M+21H]^{21+}$; 888.2 $[M+22H]^{22+}$; 849.7 $[M+23H]^{23+}$. **(C)** Analytical HPLC trace of crude reaction mixture after ligation-deselenization (0 to 15% B over 5 min, then 15 to 55% B over 40 min, 0.1% v/v TFA, λ= 214 nm); **(D)** Analytical HPLC trace of purified **Hsp27 (*p*Thr174) (12)**, R_t 23.9 min

(0 to 15% B over 5 min, then 15 to 55% B over 40 min, 0.1% v/v TFA, λ = 214 nm); **(E)** ESI-MS of pure **12**; Calculated Mass: [M+13H]$^{13+}$: 1759.6; [M+14H]$^{14+}$: 1634.0; [M+15H]$^{15+}$: 1525.2; [M+16H]$^{16+}$: 1429.9; [M+17H]$^{17+}$: 1345.8; [M+18H]$^{18+}$: 1271.1; [M+19H]$^{19+}$: 1204.3; [M+20H]$^{20+}$: 1144.1; [M+21H]$^{21+}$: 1089.7; [M+22H]$^{22+}$: 1040.2; [M+23H]$^{23+}$: 995.0; [M+24H]$^{24+}$: 953.6; [M+25H]$^{25+}$: 915.5; [M+26H]$^{26+}$: 880.3; [M+27H]$^{27+}$: 847.8. Mass Found (ESI+) 1759.8 [M+13H]$^{13+}$; 1633.7 [M+14H]$^{14+}$; 1525.0 [M+15H]$^{15+}$; 1429.7 [M+16H]$^{16+}$; 1345.6 [M+17H]$^{17+}$; 1271.4 [M+18H]$^{18+}$; 1204.5 [M+19H]$^{19+}$; 1144.3 [M+20H]$^{20+}$; 1089.9 [M+21H]$^{21+}$; 1040.3 [M+22H]$^{22+}$; 995.1 [M+23H]$^{23+}$; 953.8 [M+24H]$^{24+}$; 915.9 [M+25H]$^{25+}$; 880.6 [M+26H]$^{26+}$; 847.7 [M+27H]$^{27+}$. ESI-MS data in panel **(E)** was collected over the entire gradient and wash cycle of the UPLC-MS; **(F)** Deconvoluted mass spectrum of **16**; **(G)** Deconvoluted mass spectrum of **12**. **Note:** The 2 mass envelopes in panels **(B)** and **(E)** and the deconvoluted masses in panels **(F)** and **(G)** correspond to the presence and absence of the N-terminal methionine (MW_{Met} = 131 Da), derived from the expressed Hsp27 segment (*vide supra*).

2. Hsp27 (*p*Ser176) (13):

Hsp27 (*p*Thr176) 13 was synthesized by subjecting **Hsp27 (1-172)-NHNH$_2$ 15** (2.0 mg, 0.103 µmol) to the selenoesterification conditions described in the general procedure, followed by the addition of the **Hsp27 (173-205, *p*Ser176) diselenide 18** (0.74 mg, 0.206 µmol) in accordance with the general procedure for ligation described above. After Et$_2$O extraction to remove residual DPDS, the reaction mixture was subjected to deselenization as described in the general procedure (60 mM TCEP, 60 mM reduced GSH, pH 6.5, 16 h, room temperature) to afford full length **Hsp27 (*p*Ser176)** (**13**) as a fluffy white solid after RP-HPLC purification (0 to 15% B over 5 min then 15 to 75% B over 70 min, 0.1% v/v TFA) and lyophilization (1.15 mg, 49% yield over 3 steps).

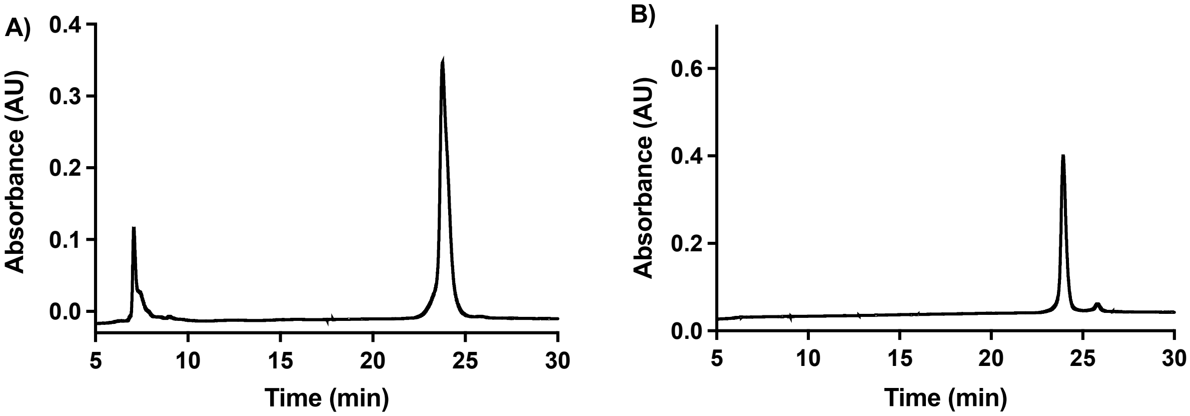

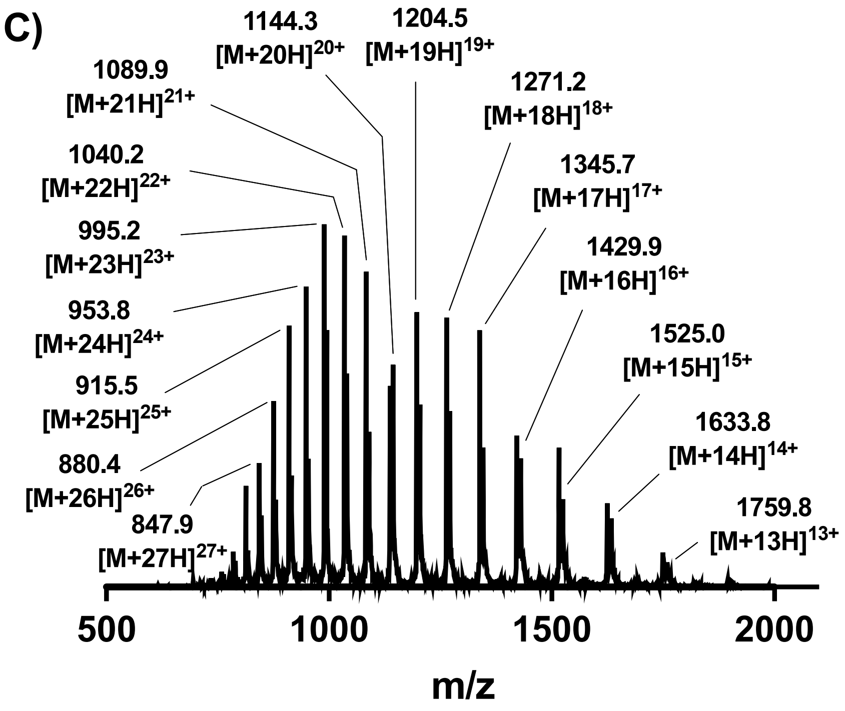

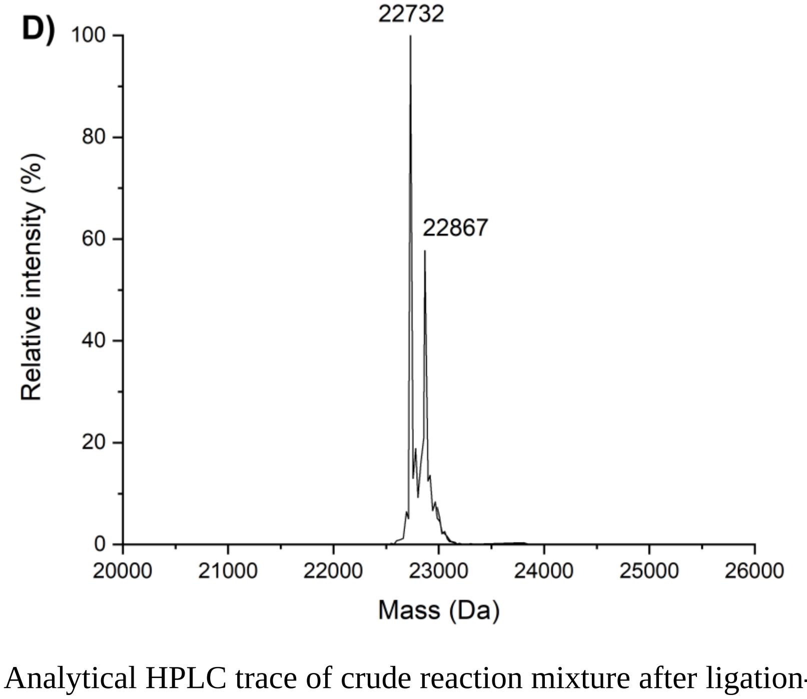

Figure S24: (A) Analytical HPLC trace of crude reaction mixture after ligation-deselenization (0 to 15% B over 5 min, then 15 to 55% B over 40 min, 0.1% v/v TFA, λ = 214 nm); **(B)** Analytical HPLC trace of purified **Hsp27 (*p*Thr176) (13)**, R_t 23.9 min (0 to 15% B over 5 min, then 15 to 55% B over 40 min, 0.1% v/v TFA, λ = 214 nm); **(C)** ESI-MS of pure **13**; Calculated Mass: $[M+13H]^{13+}$: 1759.6; $[M+14H]^{14+}$: 1634.0; $[M+15H]^{15+}$: 1525.2; $[M+16H]^{16+}$: 1429.9; $[M+17H]^{17+}$: 1345.8; $[M+18H]^{18+}$: 1271.1; $[M+19H]^{19+}$: 1204.3; $[M+20H]^{20+}$: 1144.1; $[M+21H]^{21+}$: 1089.7; $[M+22H]^{22+}$: 1040.2; $[M+23H]^{23+}$: 995.0; $[M+24H]^{24+}$: 953.6; $[M+25H]^{25+}$:

915.5; $[M+26H]^{26+}$: 880.3; $[M+27H]^{27+}$: 847.8. Mass Found (ESI+) 1759.8 $[M+13H]^{13+}$; 1633.8 $[M+14H]^{14+}$; 1525.0 $[M+15H]^{15+}$; 1429.9 $[M+16H]^{16+}$; 1345.7 $[M+17H]^{17+}$; 1271.2 $[M+18H]^{18+}$; 1204.5 $[M+19H]^{19+}$; 1144.3 $[M+20H]^{20+}$; 1089.9 $[M+21H]^{21+}$; 1040.2 $[M+22H]^{22+}$; 995.2 $[M+23H]^{23+}$; 953.8 $[M+24H]^{24+}$; 915.5 $[M+25H]^{25+}$; 880.4 $[M+26H]^{26+}$; 847.9 $[M+27H]^{27+}$. ESI-MS data was collected over the entire gradient and wash cycle of the UPLC-MS; **(D)** Deconvoluted mass spectrum of **13**. **Note:** The 2 mass envelopes in panel **(C)** and the deconvoluted masses in panel **(D)** correspond to the presence and absence of the N-terminal methionine (MW_{Met} = 131 Da), derived from the expressed Hsp27 segment (*vide supra*).

3. Hsp27 (*p*Ser199) (14):

Hsp27 (*p*Thr199) 14 was synthesized by subjecting **Hsp27 (1-172)-NHNH$_2$ 15** (2.0 mg, 0.103 µmol) to the selenoesterification conditions described in the general procedure, followed by the addition of the **Hsp27 (173-205, *p*Ser199) diselenide 19** (0.74 mg, 0.206 µmol) in accordance with the general procedure for ligation described above. After Et_2O extraction to remove residual DPDS, the reaction mixture was subjected to deselenization as described in the general procedure (60 mM TCEP, 60 mM reduced GSH, pH 6.5, 16 h, room temperature) to afford full length **Hsp27 (*p*Ser199)** (**14**) as a fluffy white solid after RP-HPLC purification (0 to 15% B over 5 min then 15 to 75% B over 70 min, 0.1% v/v TFA) and lyophilization (1.0 mg, 42% yield over 3 steps).

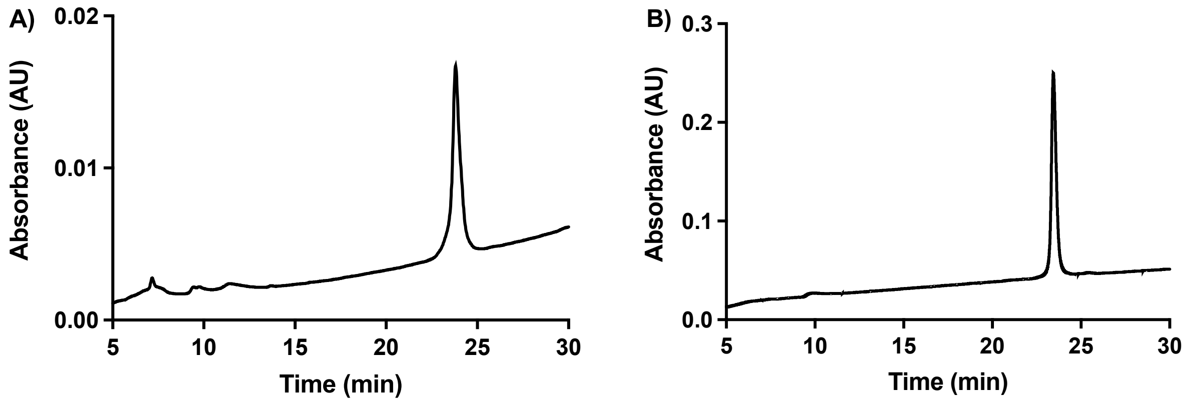

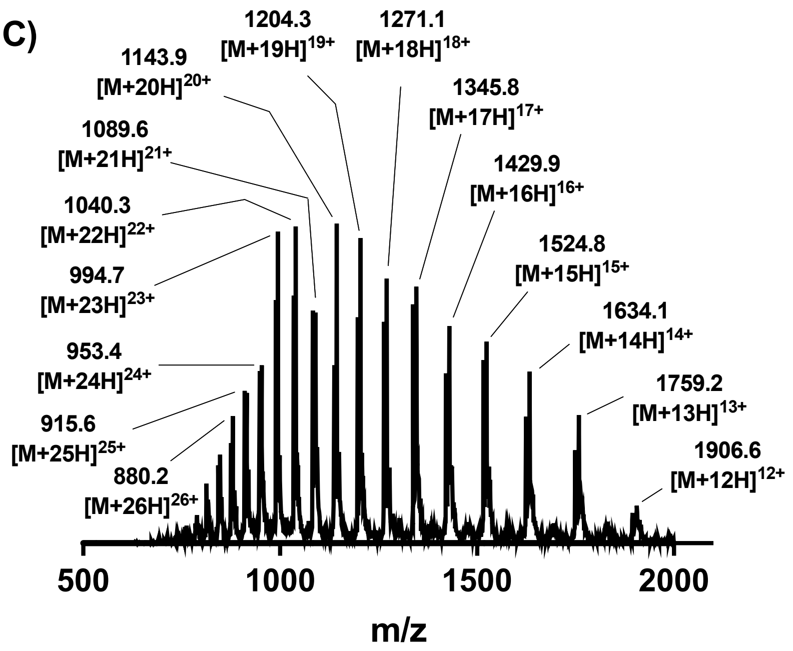

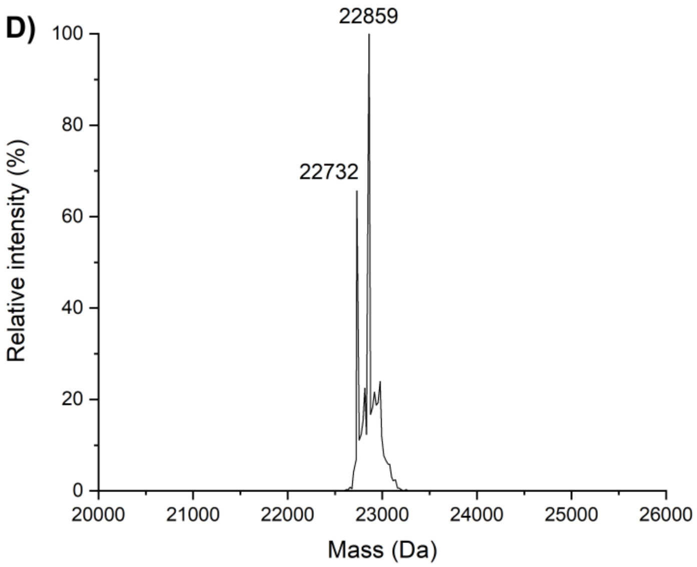

Figure S25: (A) Analytical HPLC trace of crude reaction mixture after ligation-deselenization (0 to 15% B over 5 min, then 15 to 55% B over 40 min, 0.1% v/v TFA, λ = 214 nm); **(B)** Analytical HPLC trace of purified **Hsp27 (*p*Thr199) (14)**, R_t 23.5 min (0 to 15% B over 5 min, then 15 to 55% B over 40 min, 0.1% v/v TFA, λ = 214 nm); **(C)** ESI-MS of pure **14**; Calculated Mass: $[M+12H]^{12+}$: 1906.2; $[M+13H]^{13+}$: 1759.6; $[M+14H]^{14+}$: 1634.0; $[M+15H]^{15+}$: 1525.2; $[M+16H]^{16+}$: 1429.9; $[M+17H]^{17+}$: 1345.8; $[M+18H]^{18+}$: 1271.1; $[M+19H]^{19+}$: 1204.3; $[M+20H]^{20+}$: 1144.1; $[M+21H]^{21+}$: 1089.7; $[M+22H]^{22+}$: 1040.2; $[M+23H]^{23+}$: 995.0; $[M+24H]^{24+}$: 953.6; $[M+25H]^{25+}$: 915.5; $[M+26H]^{26+}$: 880.3. Mass Found (ESI+) 1906.6 $[M+12H]^{12+}$; 1759.2 $[M+13H]^{13+}$; 1634.1 $[M+14H]^{14+}$; 1524.8 $[M+15H]^{15+}$; 1429.9 $[M+16H]^{16+}$; 1345.8 $[M+17H]^{17+}$; 1271.1 $[M+18H]^{18+}$; 1204.3 $[M+19H]^{19+}$; 1143.9 $[M+20H]^{20+}$; 1089.6

$[M+21H]^{21+}$; 1040.3 $[M+22H]^{22+}$; 994.7 $[M+23H]^{23+}$; 953.4 $[M+24H]^{24+}$; 915.6 $[M+25H]^{25+}$; 880.2 $[M+26H]^{26+}$. ESI-MS data was collected over the entire gradient and wash cycle of the UPLC-MS; **(D)** Deconvoluted mass spectrum of **14**. **Note:** The 2 mass envelopes in panel **(C)** and the deconvoluted masses in panel **(D)** correspond to the presence and absence of the N-terminal methionine (MW_{Met} = 131 Da), derived from the expressed Hsp27 segment (*vide supra*).

Folding and CD spectra of Hsp27 phosphoforms:

The refolding of all Hsp27 variants was carried out *via* overnight dialysis against a chaotropic agent free buffer. Briefly, an aliquot of a lyophilized Hsp27 sample (approx. 150 µg) was dissolved into 100 µL of 40 mM HEPES·KOH, pH 7.5 buffer containing 6 M guanidine hydrochloride followed by dialyzing it overnight into 40 mM HEPES·KOH, pH 7.5 buffer without guanidine hydrochloride. Refolded Hsp27 variants were buffer exchanged into 10 mM sodium phosphate buffer, pH 7.2 by Zeba™ Spin Desalting Columns (7K MWCO, 0.5 ml, Thermo Fisher Scientific). A final protein concentration of 0.1 mg/mL (NanoDrop, see above) in a micro cuvette (1 mm path length, Hellma Analytics, Germany) was used for each measurement.

CD spectra were recorded using a Chirascan Plus CD-spectrophotometer (Applied Photophysics, United Kingdom). CD spectra were recorded at 25 °C from 200 to 260 nm in 1 nm steps. For each spectrum, 10 measurements were averaged and the background (buffer only) was subtracted. The raw data were exported from Pro-Data software as CSV and further processed using OriginPro.

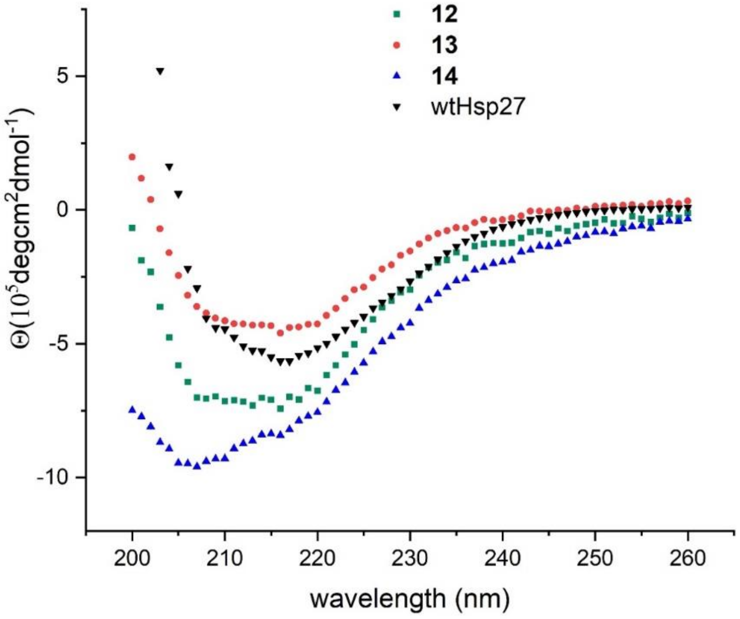

Figure S26: CD spectra of phosphorylated Hsp27 variants **12-14** compared to recombinantly produced Hsp27.

Chaperone assay for Hsp27 phosphoforms:

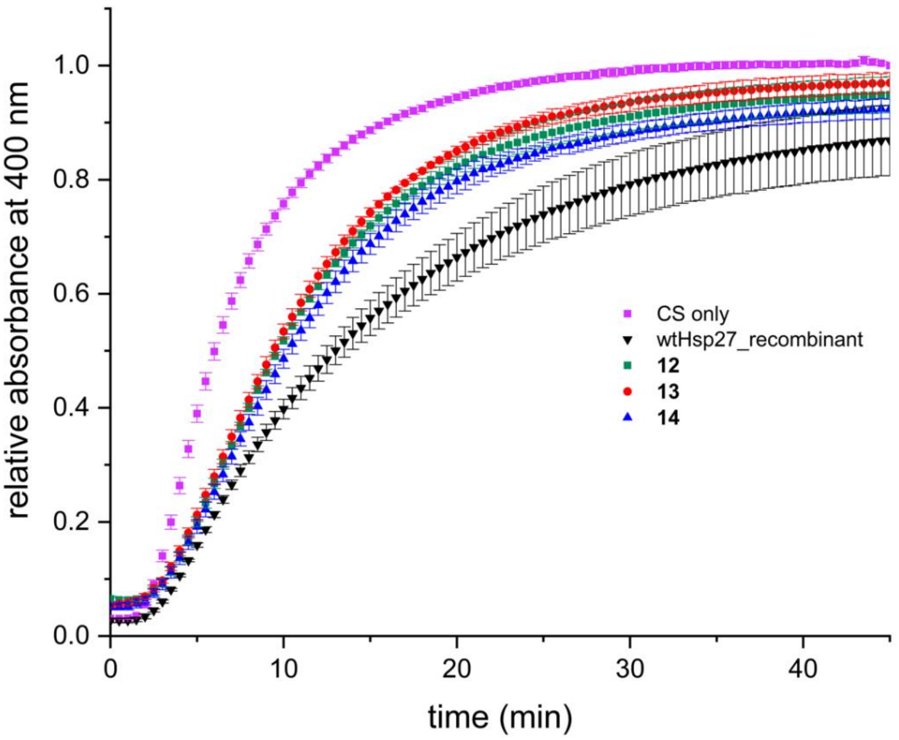

Figure S27: Aggregation assay with citrate synthase (CS) in the presence of phosphorylated Hsp27 variants **12-14** and recombinantly produced Hsp27.

The assay was performed according to the literature method with minor modifications.[7] Citrate Synthase (CS) from porcine heart was purchased from Sigma-Aldrich (Taufkirchen, Germany) as an ammonium sulfate suspension then centrifuged to remove most of the ammonium sulfate salts and dialyzed against the storage buffer (50 mM Tris·HCl, 2 mM EDTA, pH 8), final concentration 20-30 µM. The accurate concentration was then determined using bicinchoninic acid (BCA) assay (MW of CS = 48,969 Da) and this stock solution was flash frozen into liquid nitrogen in small aliquots (200-500 µL) and stored at -80 °C. Amorphous aggregation of CS was monitored via measuring the absorbance at 400 nm in a SAFAS UVmc2 double-beam UV-Vis spectrophotometer equipped with a temperature controlled multi-cell holder (SAFAS, Monaco) in 700 µL quartz cuvettes (Hellma Analytics, Germany), 600 µL final volume in triplicate. CS stock solution (obtained as above) was diluted with 40 mM HEPES·KOH (pH 7.5) to a final concentration of 2 µM, and the resulting solution was used as such (control) or treated with Hsp27 variants (0.45 µM final concentration) followed by incubation at 45 °C while measuring the absorbance at 400 nm over 45 minutes (600 µL final volume in triplicate). Prior to the addition, all Hsp27 variants (unmodified recombinant material and synthetic phosphorylated variants as lyophilized powders) were dissolved into 6 M guanidine hydrochloride in 40 mM HEPES·KOH (pH 7.5) buffer to a

concentration of approx. 1.5 mg/mL, followed by dialysis against 40 mM HEPES·KOH (pH 7.5) buffer, overnight. The accurate concentration of these primary stocks and that of CS were determined *via* BCA assay and another stock of all Hsp27 samples of concentration 1 mg/mL was prepared. A baseline correction employing only the assay buffer (40 mM HEPES·KOH, pH 7.5) was also performed. The raw data were exported from SAFAS software as a Microsoft Excel worksheet and processed using Microsoft Excel and OriginPro. The results were expressed as average relative UV absorbance at 400 nm, where relative absorption at 400 nm = (absorption at 400 nm)/(maximal absorption at 400 nm by aggregating CS in the absence of a chaperone).

^{1}H and ^{13}C NMR Spectra (building blocks S1 and S2):

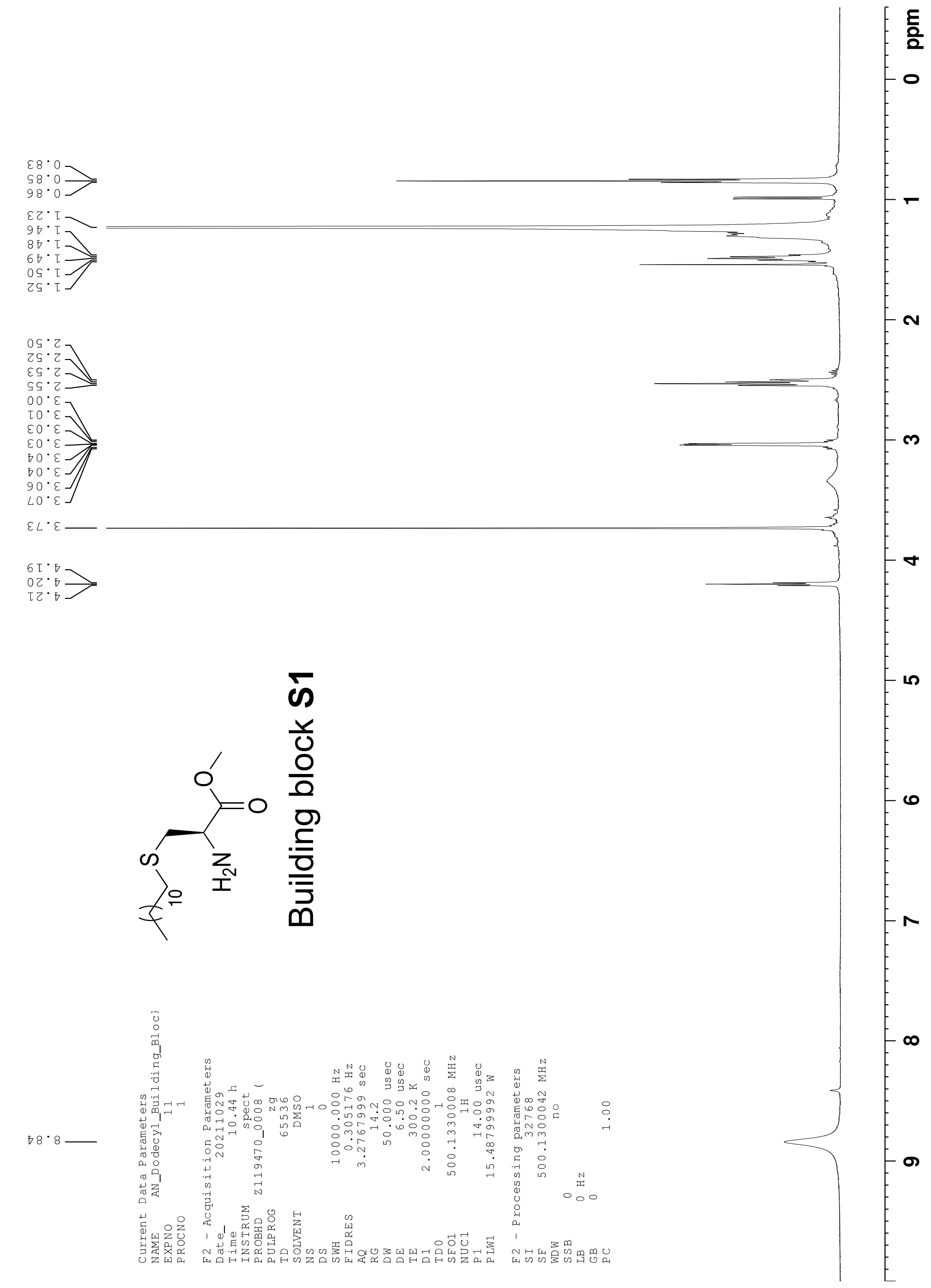

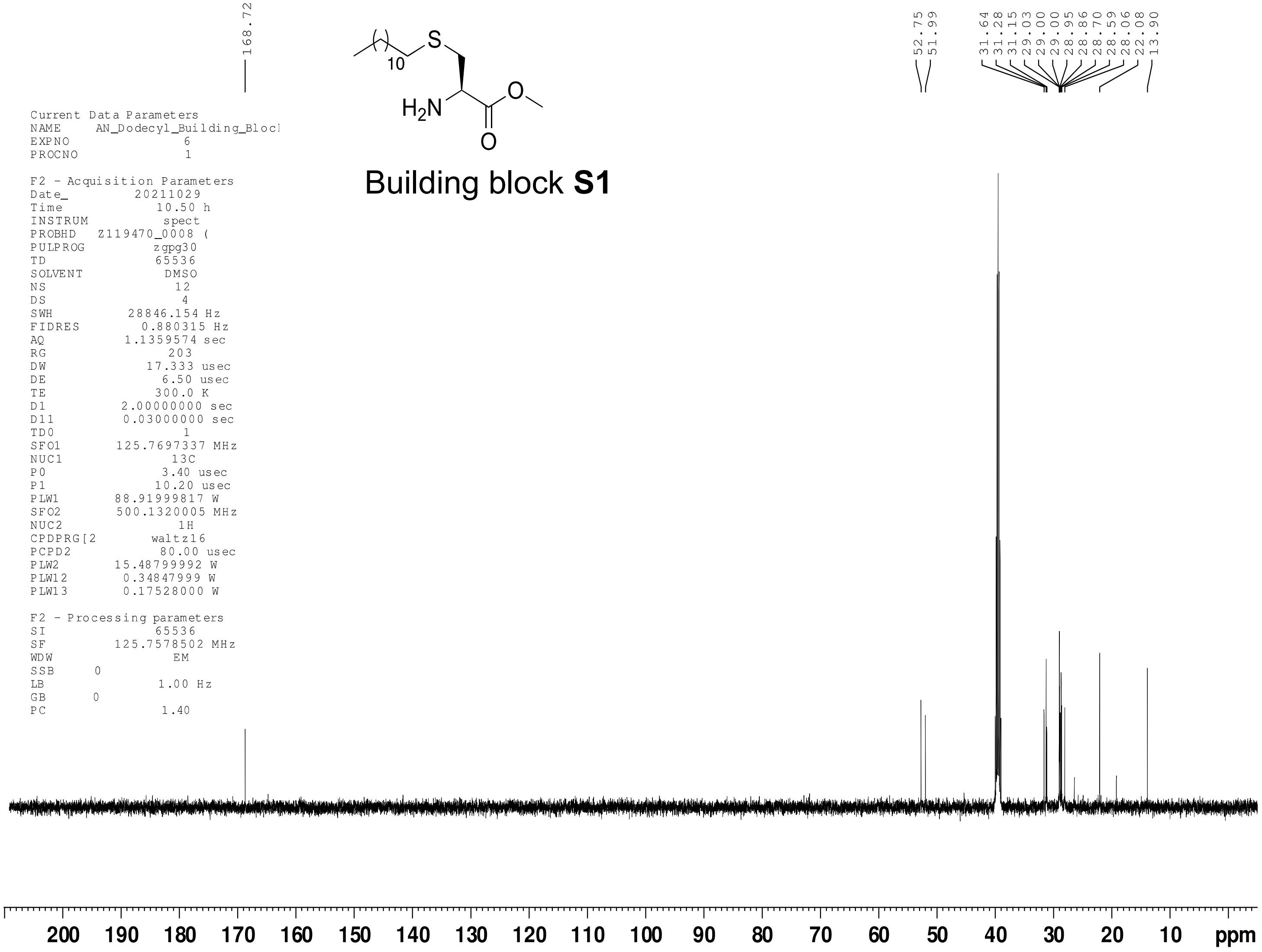

Building block **S1**

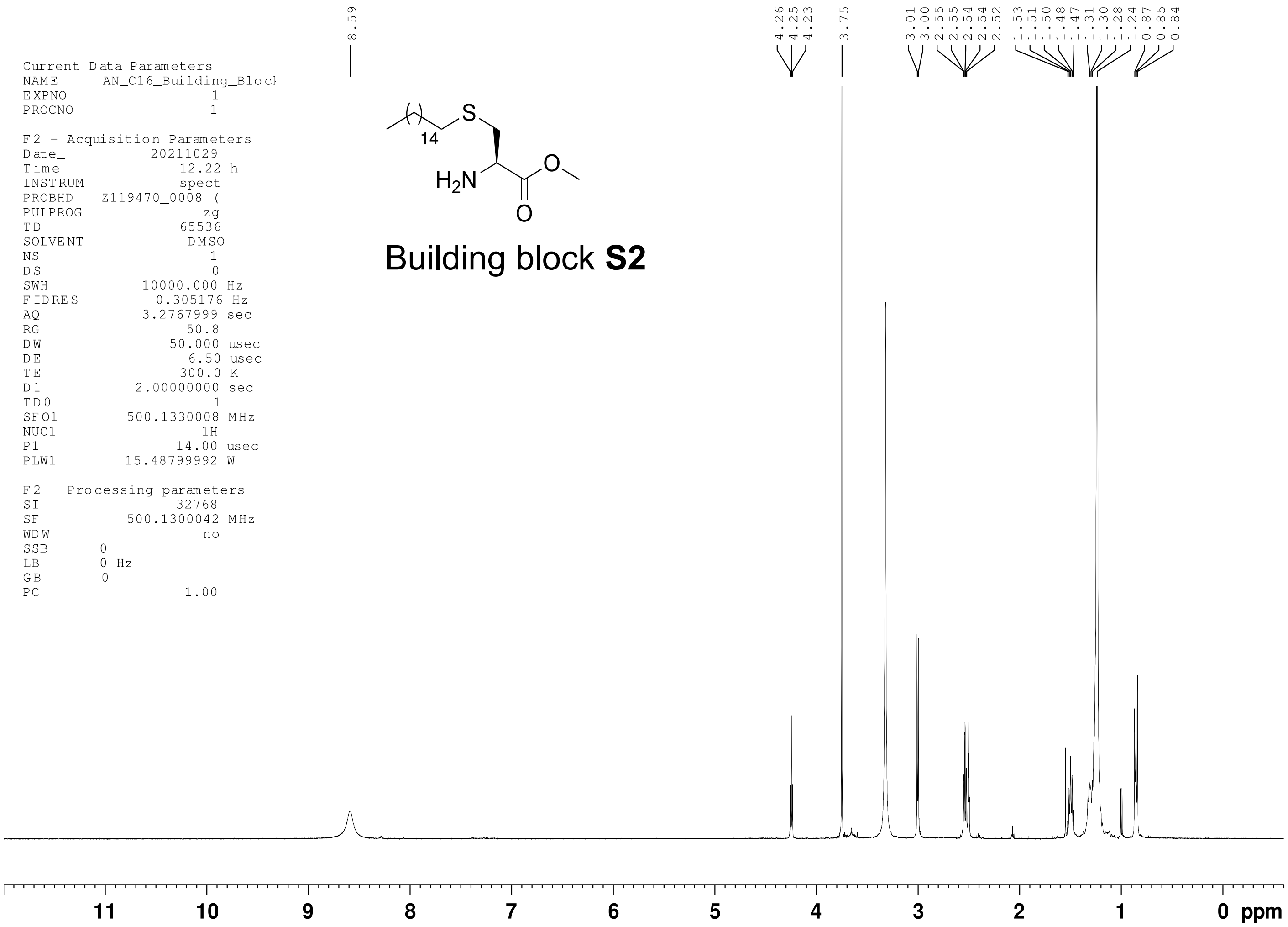

Building block **S2**

```
urrent Data Parameters
AME       AN_C16_Building_Bloc
XPNO                    2
ROCNO                   1

2 - Acquisition Parameters
ate_            20211029
ime                12.43 h
NSTRUM             spect
ROBHD   Z119470_0008 (
ULPROG           zgpg30
D                 65536
OLVENT             DMSO
S                   363
S                     4
WH            28846.154 Hz
IDRES          0.880315 Hz
Q             1.1359574 sec
G                   203
W                17.333 usec
E                  6.50 usec
E                 300.0 K
1            2.00000000 sec
11           0.03000000 sec
D0                   25
FO1         125.7697337 MHz
UC1                 13C
0                  3.40 usec
1                 10.20 usec
LW1         88.91999817 W
FO2         500.1320005 MHz
UC2                  1H
PDPRG[2         waltz16
CPD2              80.00 usec
LW2         15.48799992 W
LW12         0.34847999 W
LW13         0.17528000 W

2 - Processing parameters
I                 65536
F           125.7578506 MHz
DW                   EM
SB       0
B                  1.00 Hz
B        0
C                  1.40
```

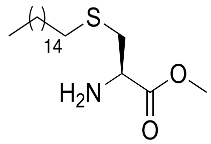

Building block **S2**

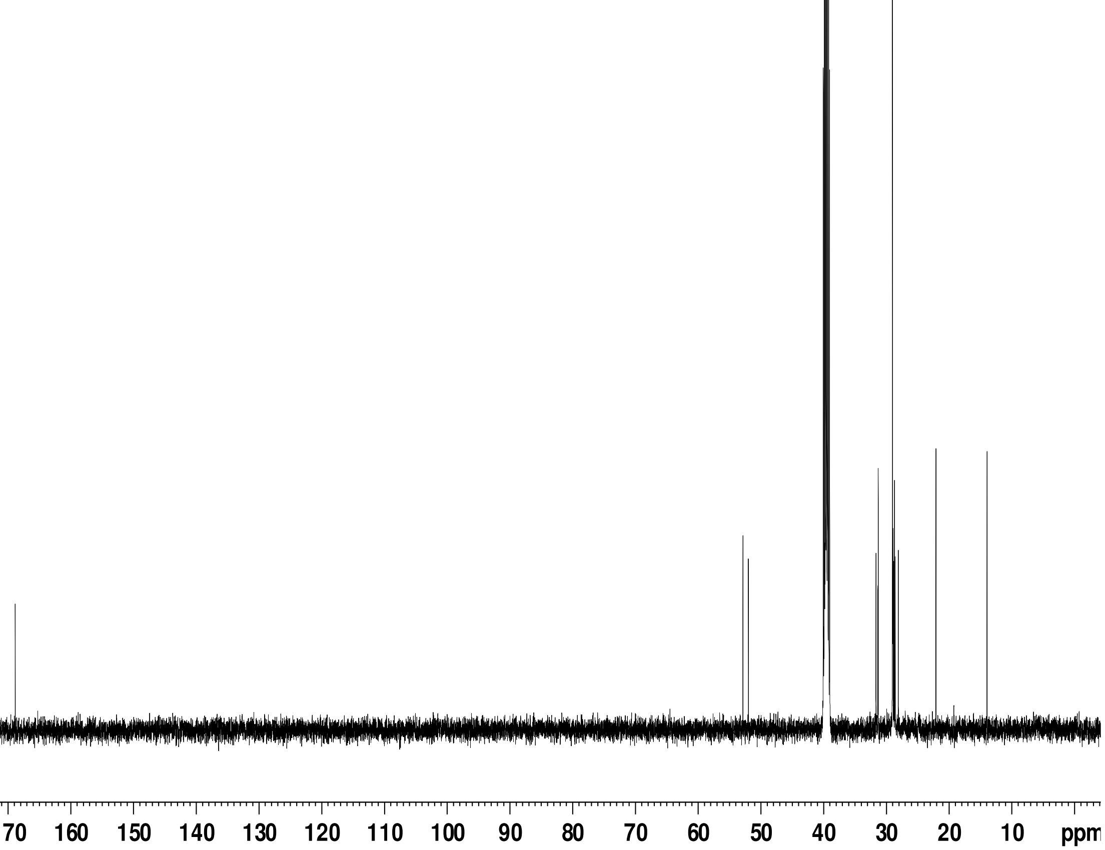

References:

[1] Z. Zhang, A. G. Marshall, *J. Am. Soc. Mass Spectrom.* **1998**, *9*, 225-233.

[2] L. R. Malins, N. J. Mitchell, S. McGowan, R. J. Payne, *Angew. Chem. Int. Ed.* **2015**, *54*, 12716-12721.

[3] J. Sayers, P. M. T. Karpati, N. J. Mitchell, A. M. Goldys, S. M. Kwong, N. Firth, B. Chan, R. J. Payne, *J. Am. Chem. Soc.* **2018**, *140*, 13327–13334.

[4] J. Jumper, R. Evans, A. Pritzel, T. Green, M. Figurnov, O. Ronneberger, K. Tunyasuvunakool, R. Bates, A. Žídek, A. Potapenko, A. Bridgland, C. Meyer, S. A. A. Kohl, A. J. Ballard, A. Cowie, B. Romera-Paredes, S. Nikolov, R. Jain, J. Adler, T. Back, S. Petersen, D. Reiman, E. Clancy, M. Zielinski, M. Steinegger, M. Pacholska, T. Berghammer, S. Bodenstein, D. Silver, O. Vinyals, A. W. Senior, K. Kavukcuoglu, P. Kohli, D. Hassabis, *Nature* **2021**, *596*, 583-589.

[5] G. Triola, L. Brunsveld, H. Waldmann, *J. Org. Chem.* **2008**, *73,* 3646-3649.

[6] M. Matveenko, E. Cichero, P. Fossa, C. F. W. Becker, *Angew. Chem. Int. Ed.* **2016**, *55*, 11397-11402.

[7] F. Narberhaus, M. Haslbeck, *Protein folding handbook*, Wiley-VCH Verlag GmbH, Weinheim, Germany, **2005**.
